# Prominent action of butyrate over β-hydroxybutyrate as histone deacetylase inhibitor, transcriptional modulator and anti-inflammatory molecule

**DOI:** 10.1038/s41598-018-36941-9

**Published:** 2019-01-24

**Authors:** Sabrina Chriett, Arkadiusz Dąbek, Martyna Wojtala, Hubert Vidal, Aneta Balcerczyk, Luciano Pirola

**Affiliations:** 1 Lyon University, Carmen Laboratory; INSERM Unit 1060; INRA, Claude Bernard Lyon-1 University, Insa-Lyon, 69921 Oullins, France; 2 Department of Molecular Biophysics, Faculty of Biology and Environmental Protection, University of Lodz, Pomorska 141/143, Lodz, 90-236 Poland

## Abstract

Butyrate and R-β-hydroxybutyrate are two related short chain fatty acids naturally found in mammals. Butyrate, produced by enteric butyric bacteria, is present at millimolar concentrations in the gastrointestinal tract and at lower levels in blood; R-β-hydroxybutyrate, the main ketone body, produced by the liver during fasting can reach millimolar concentrations in the circulation. Both molecules have been shown to be histone deacetylase (HDAC) inhibitors, and their administration has been associated to an improved metabolic profile and better cellular oxidative status, with butyrate inducing PGC1α and fatty acid oxidation and R-β-hydroxybutyrate upregulating oxidative stress resistance factors FOXO3A and MT2 in mouse kidney. Because of the chemical and functional similarity between the two molecules, we compared here their impact on multiple cell types, evaluating i) histone acetylation and hydroxybutyrylation levels by immunoblotting, ii) transcriptional regulation of metabolic and inflammatory genes by quantitative PCR and iii) cytokine secretion profiles using proteome profiling array analysis. We confirm that butyrate is a strong HDAC inhibitor, a characteristic we could not identify in R-β-hydroxybutyrate *in vivo* nor *in vitro*. Butyrate had an extensive impact on gene transcription in rat myotubes, upregulating PGC1α, CPT1b, mitochondrial sirtuins (SIRT3-5), and the mitochondrial anti-oxidative genes SOD2 and catalase. In endothelial cells, butyrate suppressed gene expression and LPS-induced secretion of several pro-inflammatory genes, while R-β-hydroxybutyrate acted as a slightly pro-inflammatory molecule. Our observations indicate that butyrate induces transcriptional changes to a higher extent than R-β-hydroxybutyrate in rat myotubes and endothelial cells, in keep with its HDAC inhibitory activity. Also, in contrast with previous reports, R-β-hydroxybutyrate, while inducing histone β-hydroxybutyrylation, did not display a readily detectable HDAC inhibitor activity and exerted a slight pro-inflammatory action on endothelial cells.

## Introduction

The interaction between gut microbiota and diet strongly influences metabolic health *via* the bacterially-dependent synthesis of numerous metabolites^1^. In particular, the gut microbiota generates the short chain fatty acids (SCFAs) acetate, propionate and butyrate^2^. Butyrate is a SCFA known to act as a histone deacetylase inhibitor (HDACi), favoring histone acetylation and thus remodeling of chromatin towards an open and transcriptionally competent state^3^. Microbiota-produced butyrate is also a primary energy source for intestinal cells, in particular colonocytes, and its absence promotes colonocyte autophagy^4^.

A key metabolite bringing a strong chemical similarity to butyrate is R-β-hydroxybutyrate, the major ketone body of the organism, produced within the mitochondria, mainly by liver. Under physiological conditions, hepatic R-β-hydroxybutyrate production is enhanced by fasting or intense exercise and provides an alternative energy source replacing the low availability of glucose^5^.

Butyrate supplementation in the diet induced an improvement in insulin sensitivity linked to augmented energy expenditure in mice^6^. The metabolic improvements brought by butyrate were due to (i) an increase of PGC1α and CPT1b (carnitine palmitoyltransferase Ib) mRNA expression in skeletal muscle, two genes involved in mitochondrial biogenesis and fatty acid metabolism respectively^6^ and (ii) improved hepatic mitochondrial efficiency^7^. In rat myotubes, we have shown that butyrate induced histone hyperacetylation, in accordance to its HDACi activity, and alleviated palmitate-induced insulin resistance *via* hyperacetylation in the proximity of IRS1 transcriptional start site, resulting in the overexpression of IRS1 mRNA and protein levels^8^.

Based on dietary administration in mice, cell culture studies and *in vitro* enzymatic assays, it was proposed that R-β-hydroxybutyrate is an HDACi^9^. The HDACi activity of R-β-hydroxybutyrate has been linked to protection against oxidative stress, *via* up-regulation of FOXO3a, catalase and mitochondrial MnSOD2^9^; as well as to anti-inflammatory effects *via* inhibition of the NLRP3 inflammasome^10^ and a promotion of autophagic flux protecting neurons from cell death^11^. At the same time, the identification of a novel transcriptionally-activating histone post-translational modification, lysine β-hydroxybutyrylation^12^, suggest that regulation of gene expression by R-β-hydroxybutyrate might also occur *via* lysine β-hydroxybutyrylation^12^.

Based on these prior findings, we made a side-by-side comparison to investigate whether both butyrate and R-β-hydroxybutyrate, due to their reported HDACi activities, have a comparable biochemical and transcriptional effects in multiple cell types and *in vitro*. We find that β-hydroxybutyrates, even at concentrations significantly higher than butyrate, do not promote histone acetylation, while promoting lysine β-hydroxybutyrylation. Furthermore, while butyrate significantly affects transcription of genes involved in metabolic control in rat myotubes, and is anti-inflammatory in human microvascular endothelial cells (HMEC-1), R-β-hydroxybutyrate has only modest effects on gene expression and exerts a slight pro-inflammatory action on endothelial cells. Our data support the strong transcriptional effects of butyrate, while calling for a reassessment of R-β-hydroxybutyrate function as HDAC inhibitor and anti-inflammatory molecule.

## Materials and Methods

### Materials and cell culture

Sodium butyrate (NaBut, catalog #303410), R-β-hydroxybutyric acid sodium salt (NaR-βOHB, catalog #298360), R-β-hydroxybutyric acid (R-βOHB, catalog #54920), S-β-hydroxybutyric acid (S-βOHB, catalog #54925) and the racemic R/S-βOHB sodium salt (Na R/S-βOHB, catalog #54965) were all purchased from Sigma-Aldrich. DL-3-aminoisobutyric acid (catalog #217794), γ-aminobutyric acid (catalog #A2129) and 4-phenylbutyric acid (4-PBA, catalog #P21005) were also purchased from Sigma-Aldrich. 500 mM stock solutions were prepared in PBS and purified using a 0.2 µM filter before use. Trichostatin A (TSA, catalog #T1952, from Sigma-Aldrich) and suberoylanilide hydroxamic acid (SAHA, catalog #SML0061 from Sigma-Aldrich) were dissolved in DMSO. For histone acetylation studies, cells were treated with the indicated concentrations of NaBut, hydroxybutyrates or the HDACi: TSA and SAHA.

L6 myoblasts, human primary myoblasts and Human Embryo Kidney 293 (HEK293) cells were cultured in DMEM, containing 4,5 g/l glucose and 10% Fetal calf Serum (FCS). At 80–90% confluence, L6 myoblasts and human primary myoblasts were differentiated into myotubes for 7 to 8 days by switching to a differentiation medium (DMEM, containing 1 g/l glucose and 2% FCS), which was replaced every second day. Human microvascular endothelial cells (HMEC-1) were cultured as previously described^13^. All cell lines used in this study were originally obtained from the ATCC (American Type Culture Collection). The experimental protocol for the obtention of muscle biopsies to generate primary human myotubes was approved by the Ethical Committees of the Hospices Civils de Lyon and performed accordingly to French legislation (Huriet Law)^14^. Study participants received an ICF (informed consent form) and gave their informed written consent to participate to the study^14^.

For gene expression studies, rat myotubes were treated with 5 mM NaBut or 20 mM R-βOHB for 24 hours, and HMEC-1 for 6 hours.

### Histone acidic extraction, Western blotting and *in vitro* HDAC activity

Cells lysates were prepared by extracting cells in a lysis buffer (20 mM Tris-HCl, 138 mM NaCl, 2.7 mM KCl, 5% (v/v), glycerol, 1 mM sodium-o-vanadate, 1% (v/v) Nonidet P-40, 5 mM EDTA, 20 mM NaF, 1:1000 proteases inhibitors cocktail (Sigma-Aldrich, P2714) pH 8.0) and centrifugation (13,000 g, 15 min, 4 °C). Histone-containing residual pellets were incubated overnight at 4 °C with 0.2 M HCl to solubilize total histones. Solubilized histones were centrifuged (13,000 g, 15 min at 4 °C). Supernatants were collected and neutralized with 1 M Tris prior to protein quantification with the Bradford reagent (BioRad). Histones were separated by 15% SDS-PAGE. Standard immunoblotting procedures and ECL detection were employed. The primary antibodies used in this study are listed in Table 1. HPR-conjugated anti-rabbit and anti-mouse secondary antibodies were from BioRad. Chemiluminescence was detected on a BioRad Chemidoc^TM^ XRS+ apparatus and images were processed using Image Lab 3.0 (BioRad).

**Table 1:** list of primary antibodies used in this study. Primary antibodies were diluted at 1:1000 to 1:2000.

*Antibody*	Catalog number	Species	Company
anti-H3K9Ac	06–942	Rabbit	Millipore Upstate Biotechnology
anti-acetyl-H3	06–599	Rabbit	Millipore Upstate Biotechnology
Anti-total-H3	Sc-8654	Goat	Santa Cruz Biotechnology
anti-H3K9/14Ac	sc-8655	Rabbit	Santa Cruz Biotechnology
anti-H3K9bhb	PTM-1250	Rabbit	PTM Bio, Chicago, IL
anti-acetyl-lysine	9441	Rabbit	Cell Signalling Technologies
anti-H2A.Zac	Ab18262	Sheep	Abcam
Anti-H3K9Me2	Ab1220	Rabbit	Abcam

*In vitro* histone deacetylase activity was performed using the HDAC activity fluorometric assay kit (Abcam, cat# ab156064) by following the manufacturer’s instructions.

### RNA extraction, reverse transcription and real-time quantitative PCR

Total RNA was isolated with TriPure Isolation Reagent (Roche) according to the manufacturer’s instructions. RNA concentration and purity was verified by optical density (OD) measurement on a Nanodrop 2000 (Thermo Fisher Scientific). cDNA synthesis was performed using the PrimescriptTM RT reverse transcription kit (Takara) according to the manufacturer’s instructions using 1 μg of total RNA in a 20 μl reaction volume. Synthesized cDNA was then brought to a 1.2 ml final volume with water. Quantitative PCR amplification was performed using a Rotor-Gene Real-Time PCR System. 5 μl of cDNA template, 5 pmoles of forward and reverse primers and 15 μl of ABsolute^TM^ QPCR SYBR Green Mix (ABgene) were added in each reaction. Reactions were incubated at 95**°**C for 10 min, followed by 40 cycles of denaturation (95**°**C for 10 sec), annealing (at gene-specific temperatures for 30 sec, see Table 2 for primer sequences and annealing temperatures) and elongation (72 °C for 30 sec). As a quality control, qPCR amplicons were analyzed by agarose gel. Analyzed genes were normalized against hypoxanthine guanine phosphoribosyltransferase (*HPRT1)* for L6 myotubes as well as for HMEC-1.

**Table 2:** sequences of primers used in this study for mRNA quantifications in L6 (upper table) and HMEC-1 cells (lower table).

Gene	Sequence	Amplicon size bp	Annealing temperature
**Rat L6 cells**.			
Hprt1	F: TTGCTGACCTGCTGGATTAC R: AGTTGAGAGATCATCTCCAC	151 pb	55 °C
Glut4	F: GGGTTTCCAGTATGTTGCGG R: CTGGGTTTCACCTCCTGCTC	183 pb	60 °C
Hk2	F: CTTCTCGTTCCCCTGCCACC R: CCATGTAGCAGGCGTTGCTG	202 pb	62 °C
Irs1	F: ACCAGAGGACCGTCAATAGC R: AAGACGTGAGGTCCTGGTTG	138 pb	60 °C
GS	F: TCAGACCCCATCTTGACCAC R: TCCATAAAGCAGCCAAAGCC	260 pb	60 °C
Gsk3β	F: TTCAGGTGGAGTTGGAAGCTGATGC R: AACACCACTGGAAGCTTGTGC	278 pb	62 °C
Hmgcs1	F: CTGGACCGCTGCTATTCTG R: TCAGGAACATCCGAGCTAGAG	157	60 °C
Eif2	F: ACCAAGGATGAGCAGCTGGA R: ATAGATGGGTCTGAGACTGC	122	58 °C
Catalase	F: CATCGGCACATGAATGGCTA R: ACCTTGGTCAGGTCAAATGG	281	60 °C
Foxo3a	F: GAGAGCAGATTTGGCAAAGG R: CCTCATCTCCACACAGAACG	182	58 °C
SOD2	F: TCATGCAGCTGCACCACAGC R: CCATTGAACTTCAGTGCAGG	138	60 °C
SOD1	F: TGAAGAGAGGCATGTTGGAG R: CCACCTTTGCCCAAGTCATC	164	58 °C
Pgc1α	F: TCCTCTGACCCCAGAGTCAC R: CTTGGTTGGCTTTATGAGGAGG	143	60 °C
CPT1b	F: AGGCAGTAGCTTTCCAGTTCAC R: CACACCCCTAAGGATACCATTC	146	60 °C
Sirt1	F: TGAGAAAATGCTGGCCTAATA R: GATAAGACGTCATCTTCAGAG	144	60 °C
Sirt3	F: CCGACATTGTGTTCTTTGG R: TCAAGCTGGCAAAAGGCTC	128	60 °C
Sirt4	F: TGACAGAGCTCCACGGATGC R: TTGGGACCTGAAAGCTCCGGAC	188	60 °C
Sirt5	F: CTGGCACCAAGAACCTTCTG R: TGGGATGCTGGCATCTTGAG	217	60 °C
COX1	F: GGTTCTTTTTTTCCGGAGTA R: ACTATACTACTACTAACAGACCG	181 bp	58 °C
Ppia	F: CAGTCTTGGCAGTGCAGAT R: ACACGCCATAATGGCACTGG	156 bp	55 °C
**Human HMEC-1 cells**			
Ccl2	F: TCTCAAACTGAAGCTCGCACT R: TGGGGCATTGATTGCATCTGG	126 bp	59 °C
IL-6	F: CTCATTCTGCCCTCGAGCC R: GACCGAAGGCGCTTGTGGA	97 bp	60 °C
IL-8	F: ACCGGAAGGAACCATCTCAC R: GGCAAAACTGCACCTTCACAC	108 bp	59 °C
GM-CSF	F: ACTTCCTGTGCAACCCAGATT R: CTCATCTGGCCGGTCTCAC	120 bp	59 °C
IL-4	F: ATCTTTGCTGCCTCCAAGAACAC R: TGCTTGTGCCTGTGGAACTGC	140 bp	61 °C
IL-1β	F: TCGCCAGTGAAATGATGGCT R: AGATTCGTAGCTGGATGCCG	138 bp	60 °C

### Genomic DNA purification

To amplify nuclear and mitochondrial genomic DNA, we used sonicated DNA prepared as previously described^15^. Briefly, cell culture medium was removed and cells were washed twice with ice-cold PBS before cross-linking for 10 minutes with 1% formaldehyde in PBS. Formaldehyde cross-linking was quenched by treatment with 0.125 M glycine, in PBS, for 10 minutes. Cells were then harvested and homogenized in 1% SDS cell lysis buffer. Chromatin was sheared by sonication, reverse cross-linked and digested with proteinase K at 62 °C for 2 hours before DNA purification on minicolums (Nucleospin Extract II, Macherey Nagel) and analysis of the eluted DNA by agarose gel. Quantitative PCR amplification of genomic DNA was performed using a Rotor-Gene Real-Time PCR System as described above.

### Cytokines and chemokines multiple parallel quantification

Cytokines and chemokines release assay was performed using the Proteome Profiler Human Angiogenesis Array Kit (R&D Systems, Minneapolis, USA, catalog code: ARY005) accordingly to the manufacturer’s instructions. HMEC-1 (1 million cells) were pre-incubated with NaBut or NaR-βOHB and after 2 hours LPS was added for the next 4 hours (total incubation time with NaBut or NaR-βOHB was 6 hours). Thereafter, cell culture supernatants were collected and incubated with a cocktail of biotinylated antibodies supplied by manufacturer. Then, samples were incubated overnight on the cytokine assay kit membrane. Following a series of washes to remove unbound material, streptavidin-horseradish peroxidase and chemiluminescent detection reagent was added to quantify the cytokines/chemokines levels on the profiler membrane.

### Statistical analysis

Experiments were performed at least three times. Values are expressed as means ± SD or SEM as indicated in the figure legends. We determined significances using one-way ANOVA test, followed by a Bonferroni’s post-hoc pairwise comparisons. Differences were considered statistically significant when *p* < 0.05.

## Results

### Butyrate, but not β-hydroxybutyrate, promotes histone acetylation. Evidence from multiple cell types and *in vitro* testing

Butyrate is an early-discovered HDAC inhibitor using human embryonic kidney 293 (HEK293) cells as a model^3^, and the same inhibitory activity, in HEK293 cells, has also been assigned more recently to β-hydroxybutyrate^9,10^. As β-hydroxybutyrate is chiral, we investigated the effect of each enantiomer and the racemic mix on histone acetylation in various cell types.

**Figure 1:**
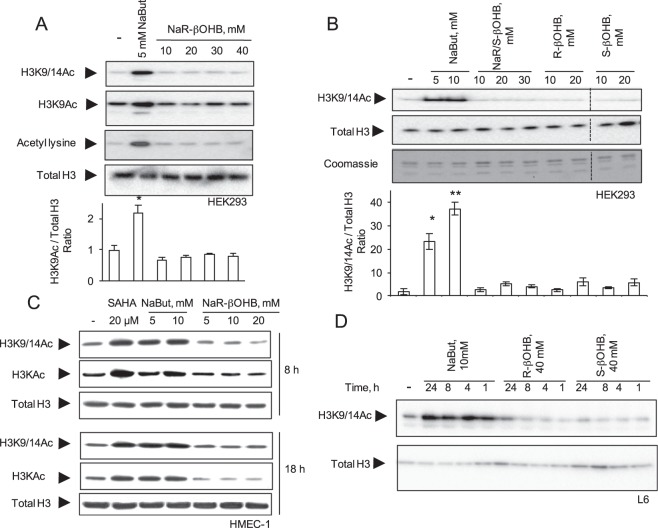
Effects of NaBut and β-hydroxybutyrate molecules on histone acetylation in multiple cell types. (**A**) HEK293 cells were incubated for 18 hours with 5 mM NaBut or increasing concentrations of NaR-βOHB (10–40 mM range). (**B**) HEK293 cells were incubated for 18 hours with NaBut, the racemic mix of β-hydroxybutyrate sodium salt (Na R/S-βOHB), R-β-hydroxybutyric acid (R-βOHB) and S-β-hydroxybutyric acid (S-βOHB) at the indicated concentrations. Separation bars indicate non-contiguous lanes on the same image acquisition (the original blots are shown in the supplementary information file). (**C**) HMEC-1 were incubated for 8 or 18 hours with SAHA, NaBut or NaR-βOHB at the indicated concentrations. (**D**) L6 myotubes were incubated with 10 mM NaBut, 40 mM R-βOHB or 40 mM S-βOHB for the indicated times. (**A**–**D**) Acid-extracted histones were immunoblotted with antibodies anti H3K9/14Ac, anti-acetyl-H3K9Ac, anti-acetyl-H3 (H3KAc), anti-acetyl lysine, anti total H3 or stained with coomassie blue as indicated. (A,B) quantification of acetyl immunoblotting signals normalized to total histone H3 content. n = 3. Error bars represent means ± SEM from 3 independent experiments. (*p < 0.05, versus control group (−) using one-way ANOVA). Quantifications for panels A (H3K9/14Ac and Acetyl lysine blots), C, D are shown in Supplementary Fig. 1.

**Figure 2:**
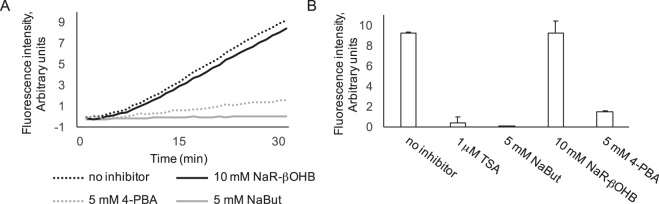
Effect of butyrates on *in vitro* HDAC activity. (**A**) HDAC activity of crude nuclear extract was measured fluorimetrically (λ_ex_ 355 nm, λ_em_ 460 nm) for 30 minutes in the presence of 10 mM NaR-βOHB, 5 mM NaBut or 5 mM 4-PBA. Fluorescence was measured every minute. One representative trace of two independent measurements is shown. (**B**) Mean fluorescence (with SD) of the two independent reactions at 30 min. 1 μM TSA was also included as positive control for inhibition.

Treatment of HEK293 cells with NaR-βOHB (in DMEM 4.5 g/l glucose, 10% FCS) for 18 hours did not increase histone acetylation as assessed with two independent antibodies to acetylated H3 (α-H3K9Ac and α-H3K9/14Ac), with anti-acetyl lysine antibodies (Fig. 1A) and with H2A.ZAc antibodies (Suppl. Fig. 2A). We next tested the effects of each β-hydroxybutyric acid stereoisoform and the racemic mixture in HEK293 cells (Fig. 1B) and primary myotubes (Suppl. Fig. 2B). Neither the racemic mixture nor the R or S enantiomers induced histone H3 acetylation after a 18 h incubation. Likewise, while treatment of HMEC-1 cells for 8 or 18 hours with either 20 μM SAHA or 10 mM NaBut promoted histone H3 hyperacetylation, administration of up to 20 mM NaR-βOHB was uneffective (Fig. 1C). Using a fluorometric *in vitro* HDAC activity assay, NaBut, TSA and 4-phenylbutyric acid (4-PBA) – a known HDACi^16^ – displayed HDAC inhibitory activity, while no HDAC inhibition was observed upon incubation with NaR-βOHB (Fig. 2).

**Figure 3:**
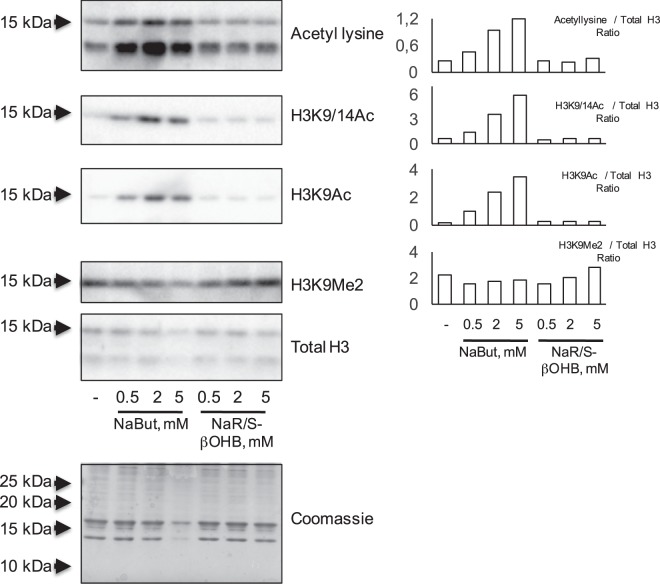
Effects of NaBut or NaR/S-β-hydroxybutyrate supplementation throughout L6 cells differentiation. L6 myoblasts were differentiated for 7 days into myotubes in the presence of increasing concentrations of NaBut or NaR/S-βOHB. Acid-extracted histones were immunoblotted with antibodies anti-acetyl lysine, anti-H3K9/14Ac, anti H3K9Ac and anti H3K9Me2. Loading was assessed by anti total histone H3 immunoblotting and coomassie staining of extracted histones. Signal quantification, relative to total H3 immunoblot signals, from representative western blots is shown on the right.

To investigate in more detail the temporal specificity in the action of β-hydroxybutyrate, L6 myotubes were incubated with 10 mM NaBut or 40 mM R-βOHB or S-βOHB in the 1–24 h range. While sodium butyrate induced a time-dependent increase of H3 acetylation, no changes were observed upon treatment with either R-βOHB or S-βOHB at any time point tested (Fig. 1D). Similarly, chronic treatment with NaBut (0.5 to 2 mM range) throghout L6 cells differentiation to myotubes (7 days) promoted histone acetylation while NaR/S-βOHB did not (Fig. 3).

**Figure 4:**
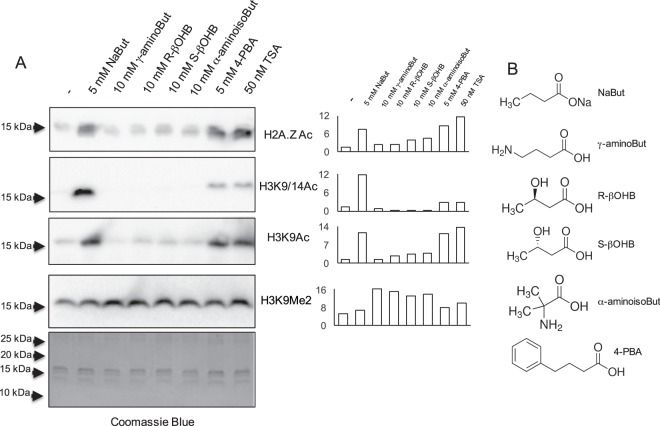
Effects of butyrate-related molecules on histone acetylation in L6 myotubes. (**A**) L6 myotubes were incubated for 18 hours with NaBut, the indicated butyrate-related molecules or TSA. Acid-extracted histones were immunoblotted with antibodies anti H2A.ZAc, anti H3K9/14Ac, anti H3K9Ac, anti H3K9Me2 or stained with coomassie blue as indicated. Signal quantification from representative western blots, relative to the coomassie blue staining signal, is shown next to each immunoblot. (**B**) Chemical formulas of NaBut and butyrate related molecules.

To obtain further insights on the specificity of butyrate-related molecules as HDAC inhibitors, L6 myotubes were treated with various butyrate-related molecules, as well as 4-PBA and trichostatin A as positive controls. As shown in Fig. 4, histone acetylation was induced by NaBut, 4-PBA and trichostatin A, but not the other butyrate-related molecules tested. Overall, our experiments based on treatment of HEK293, HMEC-1, L6 myotubes and human primary myotubes, with butyrate or butyrate-related molecules followed by immunoblotting detection with various antibodies directed to acetylated histones or acetyl-lysine do not support the action of β-hydroxybutyrate as a molecule promoting histone acetylation in cultured mammalian cells. Furthermore, *in vitro* HDAC activity was not inhibited by NaR-βOHB.

### Induction of histone β-hydroxybutyrylation by β-hydroxybutyrate and HDAC inhibitors

**Figure 5:**
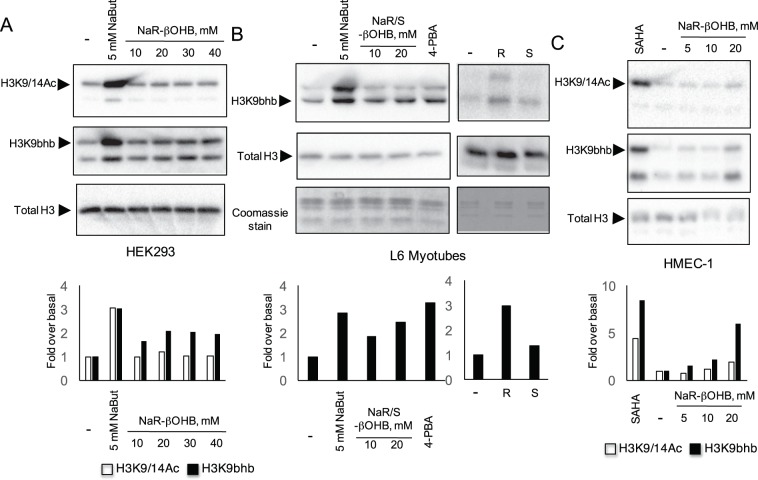
β-hydroxybutyrate and HDAC inhibitors induce histone β-hydroxybutyrylation. HEK293 cells (**A**), L6 myotubes (**B**) and HMEC-1 (**C**) were incubated for 18 hours with NaR-βOHB or the HDAC inhibitors NaBut, 4-PBA or SAHA as indicated. Acid-extracted histones were immunoblotted with antibodies anti H3K9/14Ac, anti β-hydroxybutyryl-histone H3 Lysine 9 (H3K9bhb) and anti total H3. (B, right panels) L6 myotubes were incubated for 18 hours with 10 mM R-βOHB (R) or 10 mM S-βOHB (S) and immunoblotted with antibodies anti H3K9bhb and anti total H3. Signal quantifications from representative western blots, relative to total H3 immunoblot signals, are shown. Empty bars: anti H3K9/14Ac, black bars: anti H3K9bhb).

Having observed that β-hydroxybutyrate does not promote histone lysine acetylation, we next tested its possible effects on histone β-hydroxybutyrylation, a newly discovered histone post-translational modification^12^. Exposure of HEK293, L6 myotubes and HMEC-1 cells to NaR-βOHB lead to an increase in hydroxybutyrylated lysine 9 of histone H3 (Fig. 5A–C). β-hydroxybutyrylation was mainly induced by the β-hydroxybutyrate R enantiomer (Fig. 5B, right panels). HDACi NaBut, 4-PBA and SAHA also induced histone hydroxybutyrylation (Fig. 5A–C).

### Divergent transcriptional effects of butyrate and β-hydroxybutyrate in L6 myotubes

#### Genes regulating metabolism

**Figure 6:**
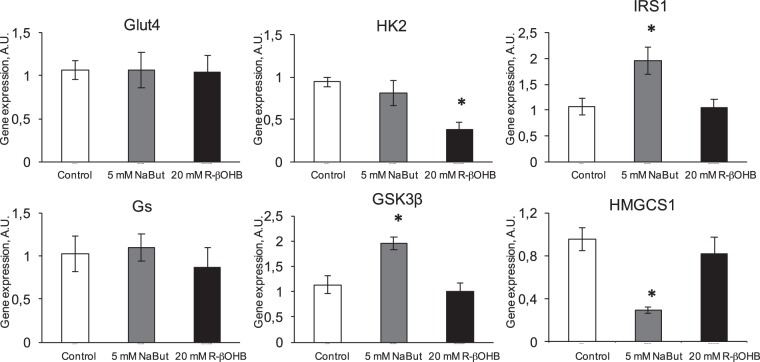
Effects of NaBut and NaR-βOHB (R-βOHB in graphs) on transcription of genes involved in insulin action. Quantification of the mRNA levels by RT qPCR for Glut4, HK2, IRS1, Gs, Gsk-3β and Hmgcs1 in L6 myotubes treated for 24 h with 5 mM NaBut (grey bars) or 20 mM NaR-βOHB (black bars). Gene expressions are normalized to the Hprt1 housekeeping gene. Error bars represent the mean ± SEM from 3 independent experiments. (*p < 0.05, versus Ctrl group using one-way ANOVA).

As both butyrate and β-hydroxybutyrate are implicated in the control of energy homeostasis, we investigated their transcriptional effects on insulin signaling genes in L6 myotubes. NaBut induced an upregulation of IRS1 and GSK3β, while downregulating the cholesterol biosynthetic gene hydroxyl-3-methylglutaryl CoA synthase 1 (HMGCS1) and having no effect on the glucose transporter GLUT4, hexokinase 2 (HK2) and glycogen synthase (GS) (Fig. 6). NaR-βOHB did not induce transcriptional changes on any of these genes, except for a HK2 downregulation (Fig. 6). As HK2 catalyses the first step of glycolysis, NaR-βOHB - dependent HK2 gene downregulation might be related to downregulation of glycolysis and a switch by the cells to the use of ketone bodies as primary energy source.

#### Upregulation of genes involved in protection from oxidative stress by NaBut

**Figure 7:**
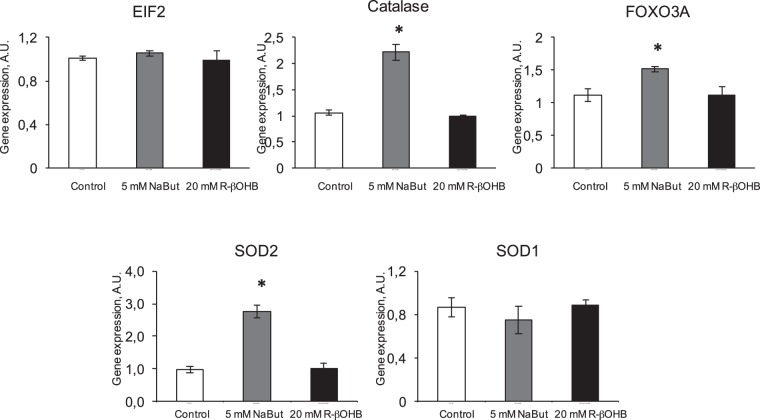
Effects of NaBut and NaR-βOHB (R-βOHB in graphs) on transcription of stress resistance and oxidative stress – protective genes. Quantification of the mRNA levels by RT qPCR for stress-resistance genes (EIF2 and FOXO3a) and oxidative stress protecting genes (catalase, SOD1, SOD2) in L6 myotubes treated for 24 h with 5 mM NaBut (grey bars) or 20 mM NaR-βOHB (black bars). Data are presented and analyzed as in Fig. 6.

Several genes conferring cellular protection against oxidative stress, including FOXO3a, mitochondrial superoxide dismutase (SOD2) and catalase were reported to be upregulated by β-hydroxybutyrate^9^. Here, we compared the effects of NaBut and NaR-βOHB on transcriptional changes on these genes. Gene expression of the stress marker EIF2 was also evaluated. EIF2 gene expression was not modified by Nabut or NaR-βOHB, indicating that neither of the two molecules induces cellular stress in L6 myotubes (Fig. 7). Catalase, FOXO3a and SOD2 – but not the cytoplasmic SOD1 - were upregulated by NaBut, while none of these genes was modulated by NaR-βOHB (Fig. 7).

#### Regulators of metabolism and healthspan

**Figure 8:**
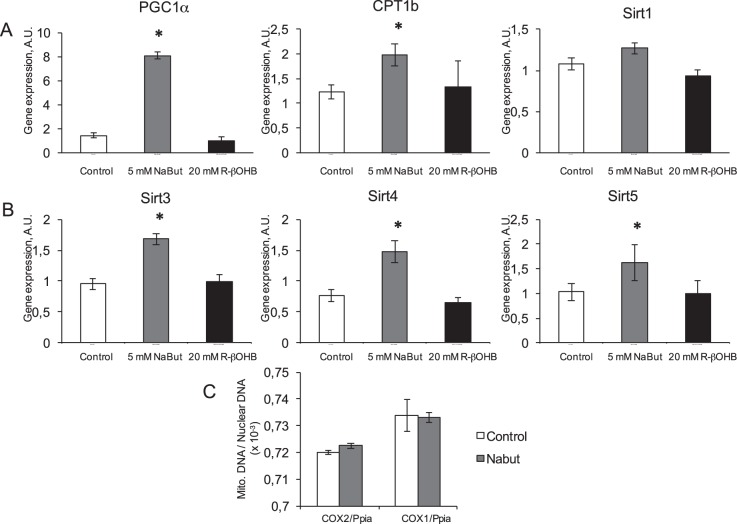
NaBut, but not NaR-βOHB (R-βOHB in graphs) affect transcription of multiple genes related to mitochondrial function. (**A**) Quantification of mRNA levels by RT qPCR for PGC1α CPT1b and SIRT1 in L6 myotubes treated for 24 h with 5 mM NaBut (grey bars) or 20 mM NaR-βOHB (black bars). (**B**) Quantification of mRNA levels of mitochondrial Sirtuins 3,4 and 5 in L6 myotubes treated for 24 h with 5 mM NaBut (grey bars) or 20 mM NaR-βOHB (black bars). (**A,B**) Data are presented and analyzed as in Fig. 6. (**C**) Mitochondrial to nuclear genome ratio as measured by amplification of two mitochondrial gene loci (Cox2 and Cox1) and a nuclear locus (on the Ppia gene) in a control or 5 mM NaBut conditions. Data are presented and analyzed as in Fig. 6.

In order to get a larger view of what are the consequences of butyrates treatment in L6 myotubes, we investigated several metabolic regulators. PGC1α, the master switch for mitochondrial biogenesis, was strongly induced by NaBut treatment, as well as expression of carnitine palmitoil transferase 1b (CPT1b), the mitochondrial enzyme catalyzing the transesterification of acyl-CoA esters and carnitine to form acylcarnitine esters to be imported within the mitochondrial matrix. On the contrary, no regulation of either gene occurred upon NaR-βOHB treatment (Fig. 8A). Sirtuins are major regulators of metabolism and ageing and, due to their action as deacetylases, might be submitted to feedback regulatory control mechanisms by HDACi. We examined the cytosolic sirtuin SIRT1 and the mitochondrial sirtuins 3–5. We found that, despite being implicated in various pathways regulating metabolism, homeostasis and aging, neither NaBut nor NaR-βOHB treatments affected SIRT1 expression (Fig. 8A). As NaBut induced PGC1α, we next evaluated on the gene expression of the three mitochondrial sirtuins SIRT 3–5. NaBut treatment significantly upregulated the SIRT 3, 4 and 5, while NaR-βOHB had no effect (Fig. 8B). Overall, NaBut upregulated several mRNAs coding for mitochondrial proteins. Thus, we next quantified mitochondrial DNA content to evaluate whether butyrate-induced gene overexpression of mitochondrially located protein is associated to an increased number of mitochondria or represents a specific accumulation of these selected genes. Using a comparative DNA dosage of nuclear and mitochondrial DNA, we determined that there is no change in mitochondrial number (Fig. 8C). Indeed, mitochondrial DNA copy number over nuclear DNA copy number did not change with NaBut treatment. Consequently, the gene overexpression we highlighted are more likely to be due to mitochondrial accumulation for these gene products rather than a higher mitochondrial number.

### Inhibitory action of butyrate and promoting action of β-hydroxybutyrate on inflammatory genes in HMEC-1

**Figure 9:**
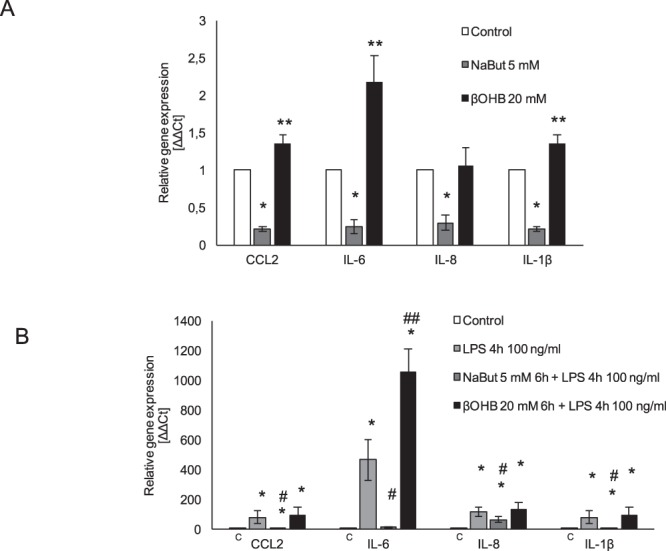
Opposite actions of NaBut and NaR-βOHB on inflammatory markers in HMEC-1. (**A**) Analysis of inflammatory gene expression in quiescent HMEC-1 cells treated with 5 mM NaBut or 20 mM NaR-βOHB for 6 h. Results are the average +/− SD of 3 independent experiments. Statistical analysis was performed using one-way ANOVA followed by a Bonferroni post-hoc test. *significantly lower than control, p < 0.05; **significantly higher than control, p < 0.05. (**B**) Analysis of inflammatory gene expression in LPS-activated HMEC-1 cells treated with 5 mM NaBut and 20 mM NaR-βOHB. Cells were incubated with butyrates for 6 hours, with addition of 100 ng/ml LPS in the last 4 hours of incubation. Results are the average +/−SD of 3 independent experiments. Statistical analysis was as in panel A. *significantly higher than control, p < 0.05; ^#^significantly lower than the LPS treatment condition, p < 0.05; ^##^significantly higher than the LPS treatment condition, p < 0.05.

**Figure 10:**
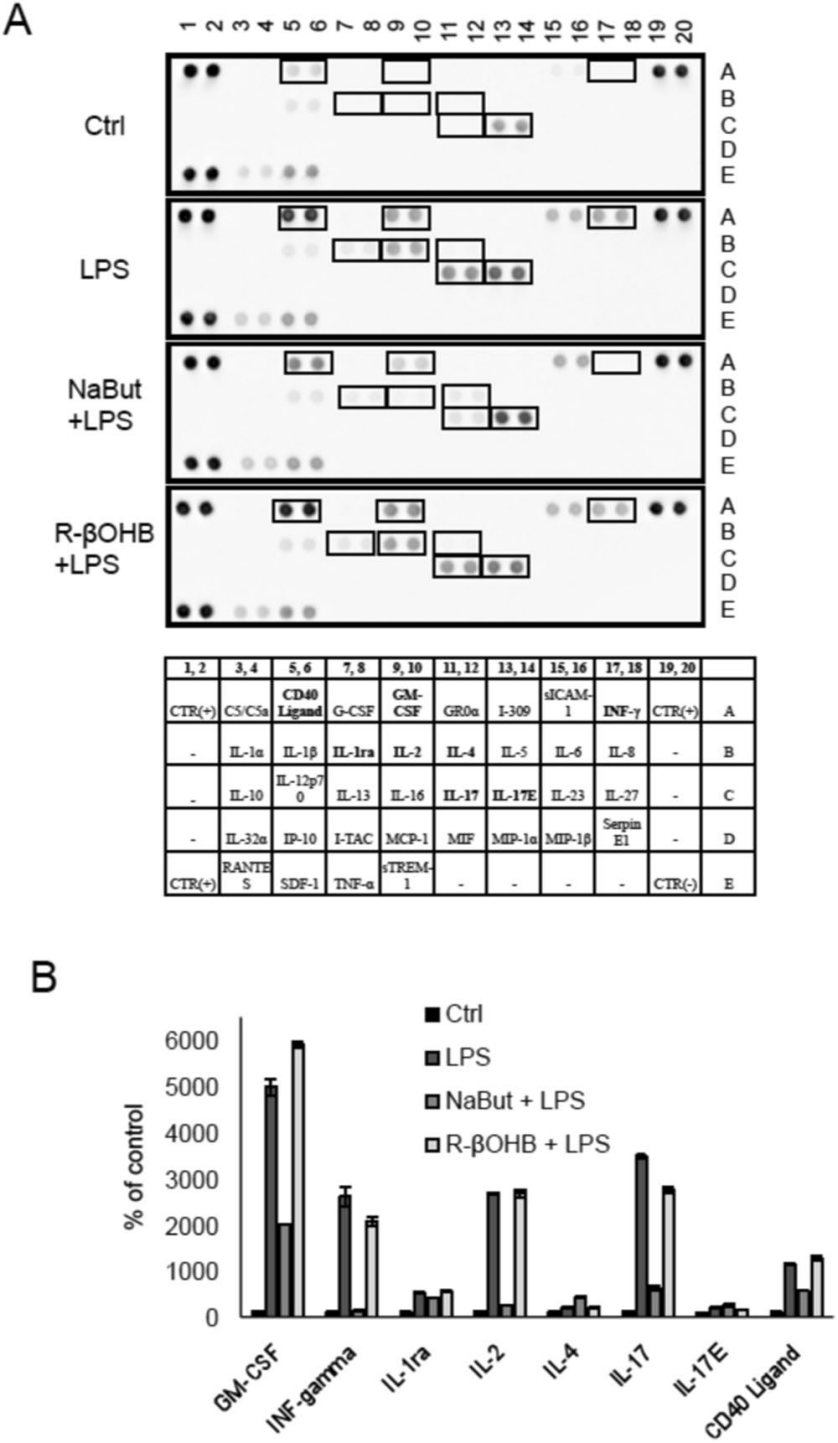
Effect of NaBut and NaR-βOHB on the release of the cytokines and chemokines in LPS-treated HMEC-1. (**A**) Secreted cytokines and chemokines in the cell culture supernatants from LPS-treated HMEC-1 cells in the presence/absence of butyrates. Cells were incubated with NaBut and NaR-βOHB for 6 hours, with addition of 100 ng/ml LPS in the last 4 hours of incubation. (**B**) The intensity of the signals of secreted factors was quantified using a CCD digital imaging system Alliance Mini HD4 (UVItec Limited, Cambridge, United Kingdom).

As butyrate and β-hydroxybutyrate have been both reported to be anti-inflammatory molecules, we compared their effect on gene expression of inflammatory genes in quiescent and LPS-stimulated endothelial cells. In quiescent HMEC-1, basal expression of inflammatory genes CCL2, IL-6, IL-8 and IL-1β was diminished by a 6 h NaBut treatment, while NaR-βOHB significantly increased CCL2, IL-6 and IL-1β gene expression (Fig. 9A), suggesting a potential pro-inflammatory action of β-hydroxybutyrate on the endothelium. Activation of HMEC-1 by LPS led to a ≈100-fold upregulation of the gene expression of CCL2, and IL-1β which was significantly decreased by NaBut, but not NaR-βOHB co-treatment. LPS-induced IL-8 gene expression was also significantly decreased by NaBut treatment. Finally, LPS-induced IL-6 gene upregulation was totally inhibited by NaBut, while NaR-βOHB treatment potentiated the LPS activatory action on IL-6 gene expression (Fig. 9B). To further investigate the effects of NaBut and NaR-βOHB, LPS-induced secretion of a panel of cytokines/chemokines was measured in the presence of absence of butyrates (Fig. 10).

LPS induced the release of a number of inflammatory mediators, including CD40-ligand, GM-CSF, IFNγ, IL-2, IL-17, and IL-17E. All of these mediators, with the exception of IL-17E, were significantly downregulated or returned to baseline upon NaBut co-treatment – pointing to an anti-inflammatory action of NaBut, while NaR-βOHB co-treatment was without effect. Furthermore, secretion of anti-inflammatory IL-4 was augmented by NaBut but not by NaR-βOHB.

Thus, on LPS-treated HMEC-1 cells, while NaBut has a clear anti-inflammatory effect, NaR-βOHB has a neutral, or slightly pro-inflammatory function.

## Discussion

β-hydroxybutyrate brings structural similarity to butyrate and has been shown to act as a HDACi^9,10^. Our previous report that butyrate, *via* its HDACi activity, ameliorates insulin signaling^8^ prompted us to evaluate in parallel the activities of butyrate and β-hydroxybutyrate as HDACi, on the rationale that β-hydroxybutyrate would be a better bioavailable HDACi as its circulating concentrations might reach millimomar levels. Treatment with the β-hydroxybutyrate racemic mix, or of each enantiomer, at concentrations up to 40 mM did not yield a significantly increased histone acetylation in for different cell types: HEK293 cells, HMEC-1, primary human myotubes and rat L6 myotubes (Figs 1, 3 and Suppl. Fig. 2). In addition, NaR-βOHB did not show *in vitro* HDAC inhibitory activity (Fig. 2). Similarly, other butyrate analogs (γ-aminobutyrate and α-aminoisobutyrate) did not act as HDACi, while 4-phenylbutyric acid, a previously reported HDACi^16^, did (Fig. 4). These observations, also supported by a recent report showing the absence of β-hydroxybutyrate – induced histone H3K18 hyperacetylation in colonic cancer cells^17^, call for a reassessment of the action of β-hydroxybutyrate as an HDACi. β-hydroxybutyrate was added to cells in the 10 to 40 mM concentration range, a sufficiently high concentration to exclude that the lack of HDAC inhibitory activity could be due to inhibitor depletion by utilization by the cells – also because glucose was present in the culture medium.

Besides a putative function as HDACi, endogenous β-hydroxybutyrate can also be a substrate to generate β-hydroxybutyryl-CoA, which serves as a histone acetyl(acyl) transferase substrate. Indeed, histone lysine β-hydroxybutyrylation has been recently shown to be a novel histone post-translational modification^12^. Therefore, we sought to identify whether multiple cell lines exposed to β-hydroxybutyrate would display increased histone H3 lysine 9 β-hydroxybutyrylation. Treatment with either R-β-hydroxybutyrate or with the racemic mixture augmented histone H3 lysine 9 β-hydroxybutyrylation (Fig. 5), with the R-β-hydroxybutyrate stereoisomer being preferentially used as substrate (Fig. 5B). Of note, also the treatment with classical HDAC inhibitors NaBut, 4-PBA and SAHA induced histone β-hydroxybutyrylation, to an even higher extent of β-hydroxybutyrate itself. Such increased β-hydroxybutyrylation by HDACi treatment likely reflects the inhibition of the deacylationof endogenously β-hydroxybutyrylated histones. This possibility is supported by several investigations demonstrating that histone deacetylases also efficiently catalyze histone deacylation, being able to remove 2-hydroxyisobutyrylation^18,19^ and crotonylation^20,21^ marks, and therefore likely also β-hydroxybutyrylation. Furthermore, Wu *et al*. showed that SAHA treatment simultaneously increased 2-hydroxyisobutyrylation, acetylation and crotonylation^19^, suggesting that increased acylation upon HDACi treatment – as we observed also for β-hydroxybutyrylation – is a general response to HDAC inhibitors. Converging data supporting our findings were recently published by Fellows *et al*., who showed that microbiota-derived butyrate and the HDACi SAHA and TSA lead to increased histone crotonylation in colonic HCT116 cells, while neither histone crotonylation or acetylation were promoted by β-hydroxybutyrate^17^.

Our data questioning the action of β-hydroxybutyrate as a clearcut HDACi, associated to the recent discovery of histone lysine β-hydroxybutyrylation as a novel histone post-translational modification^12^, calls for a comparative re-assessment of the transcriptional and biological effects on of butyrate versus β-hydroxybutyrate. As β-hydroxybutyrate is a central metabolic fuel, we focused our investigation on myotubes, as skeletal muscle is the quantitatively main tissue governing glucose disposal. In addition, as the crosstalk between whole body metabolism and endothelial metabolism are strongly related - with metabolic unbalances as insulin resistance, obesity or diabetes being associated with endothelial dysfunction and cardiovascular disease - we assessed the effects of β-hydroxybutyrate on the endothelial inflammatory response.

Butyrate has a direct effect on insulin signaling and improves L6 myotubes insulin sensitivity through an upregulation of IRS1, due to an increased histone acetylation in the proximity of the IRS1 transcriptional start site^8^. Here, we showed that, on the contrary, β-hydroxybutyrate did not affect gene expression of insulin signaling genes, as compared to butyrate with the only exception of hexokinase 2 downregulation (Fig. 6). As β-hydroxybutyrate is a fuel metabolite produced under starving and glucose deprivation, downregulation of hexokinase 2 by β-hydroxybutyrate may reflect the metabolite’s capability of downregulating the less needed glycolytic pathway. In accordance, it has also been shown that β-hydroxybutyrate causes an inhibition of insulin signaling in oxidative muscles via a 50% inhibition of insulin-mediated phosphorylation of protein kinase B^22^.

Butyrate upregulates catalase mRNA. Catalase participates in cell defense, especially in response to an oxidant injury. Dysregulation of catalase expression occurs in several diseases, including cancer^23^. In L6 myotubes, we observed catalase, as well as SOD2, gene overexpression upon butyrate treatment, while β-hydroxybutyrate did not induce either gene, as conversely observed in kidney tissue from mice implanted with osmotic pumps delivering β-hydroxybutyrate^9^.

Gene expression of the transcriptional co-activators FOXO3a and PGC-1α was increased by butyrate treatment (Figures 7 and 8), and their over-expression is linked to protection from oxidative stress protection^24,25^. Additionally, we found a number of mitochondrial genes to be upregulated by butyrate, but not β-hydroxybutyrate, including the sirtuins isoforms 3, 4, 5, SOD2 and CPT1b (Carnitine palmitoyltransferase Ib). All these upregulated genes are implicated in mitochondrial function and protection from oxidative stress and are potentially linked to the master upstream regulator PGC-1α^25^. A decrease in mitochondrial mass and PGC-1α has been found in type II diabetic patients, which was associated with decreased oxygen consumption^26^. In our study, butyrate treatment strongly upregulated PGC-1α (yet with no change in mitochondrial DNA amount) as also observed in animal models in which butyrate supplementation improved insulin sensitivity^6^.

Ketone bodies, of which the quantitatively most represented is β-hydroxybutyrate, are essential alternative fuel molecules used by the body during caloric restriction^27^. The systemic anti-inflammatory state of the organism during caloric restriction has been associated to a β-hydroxybutyrate - mediated inhibition of the NLRP3 inflammasome in bone marrow derived macrophages^10^ and circulating neutrophils^28^. Here, in endothelial HMEC-1 cells, we failed to show a clear anti-inflammatory action of β-hydroxybutyrate, while butyrate significantly inhibited both basal and LPS-induced gene expression of CCL2, IL-6, IL-8 and IL-1β (Fig. 9). Furthermore, using a cytokine array we observed that while butyrate was able to decrease the secretion of several LPS-induced pro-inflammatory factors, including GM-CSF, IFNγ, IL-1Ra, IL-2 and IL-17, and concomitantly increase anti-inflammatory IL-4 secretion; β-hydroxybutyrate was ineffective (Fig. 10). For some of these factors (GM-CSF, IL-1Ra and IL-4), gene expression analysis paralleled the secretory pattern (data not shown), while for others gene expression analysis fell below the detection threshold. Overall, while on neutrophils and macrophages β-hydroxybutyrate has been reported to act as an anti-inflammatory molecule, its effects on the endothelium are likely to be minor. A large body of evidence accumulated over the last 20 years confirms that butyrate is an inhibitor of NF-κB and its use prevents the activation of downstream signaling cascades regulated *via* the transcription factor. The inhibitory action of butyrate was proven on different models, form cell culture through animal models and human biopsies^29–32^. All these papers converge on the notion that butyrate treatment results in decreased production of ROS and reduced inflammation, which is coherent with our observations showing that butyrate alleviates inflammatory responses induced by LPS by decreasing the expression of proinflammatory molecules. On the contrary, we did not detect an alleviation of the expression of proinflammatory molecules in β-hydroxybutyrate – treated HMEC-1. Consistently with our observations, Shi *et al*.; showed that β-hydroxybutyrate can induce cattle hepatocyte inflammatory injury and oxidative stress through the NF-κB signaling pathway, by increasing the expression of TNF-α, IL-6 and IL-1β. In this model, co-treatment of cells with β-hydroxybutyrate and the antioxidant N-acetylcysteine markedly reduced the extent of the inflammation^33^.

Ketogenesis, with the endogenous production of β-hydroxybutyrate and other ketone bodies, is an evolutionarily conserved alternative metabolic strategy to provide an energy source during starving, neonatal life of prolonged physical effort. Yet, on the other side, ketoacidosis – with β-hydroxybutyrate levels rising to up to 20 mM - is a serious complication of diabetes. While, on the one side, ketogenesis as a result of caloric restriction or ketogenic diets has potential benefits on metabolic health, longevity and healthspan^34,35^, on the other side possible long-term detrimental effects should not be discounted, as an 8-month ketogenic diet has been shown to have detrimental effects on skeletal muscle physiology^36^.

Albeit part of the current literature indicates that induction of ketogenesis and/or β-hydroxybutyrate supplementation might be potentially beneficial in a clinical translational setting, caution must be exerted as β-hydroxybutyrate might not pleiotropically act as anti inflammatory molecule, HDACi and as protective agent against oxidative stress.

## Supplementary information

Supplementary material

## Data Availability

All the original data acquisition of western blots is included in a Supplementary Information file.
