# Supplementary material for: Prominent action of butyrate over β-hydroxybutyrate as histone deacetylase inhibitor, transcriptional modulator and anti-inflammatory molecule
Source: Sci Rep. 2019 Jan 24;9:742. doi: 10.1038/s41598-018-36941-9 (PMC6346118; doi:10.1038/s41598-018-36941-9)
Supplement: Supplementary file 1 — Supplementary material [file 41598_2018_36941_MOESM1_ESM.pdf]

Prominent action of butyrate over β-hydroxybutyrate as histone deacetylase inhibitor, transcriptional modulator and anti-inflammatory molecule

Sabrina Chriett[1], Arkadiusz Dąbek[2], Martyna Wojtala[2], Hubert Vidal[1], Aneta Balcerczyk*[2], Luciano Pirola*[1]

[1] Lyon University, Carmen Laboratory; INSERM Unit 1060; INRA, Claude Bernard Lyon-1 University, Insa-Lyon; 69921 Oullins, France.

[2] Department of Molecular Biophysics, Faculty of Biology and Environmental Protection, University of Lodz, Pomorska 141/143, Lodz, 90-236, Poland.

* AB and LP equally contributed to this work

Address correspondence to Luciano Pirola, INSERM U1060, 165 Ch. du Grand Revoyet - BP12, 69921 Oullins, France, email: luciano.pirola@univ-lyon1.fr, tel. ++33 4 26 23 59 28

Running title: HDACi actions of butyrate and hydroxybutyrate

Data availability statement: All the original data acquisition of western blots is included in a Supplementary Information file.

Abbreviations: HDAC: histone deacetylase; HDACi: histone deacetylase inhibitor; NaBut: sodium butyrate; PTM: post-translational modification; R-βOHB: R-β-hydroxybutyric acid. SCFAs: short chain fatty acids.

Supplementary figure legends

Supplementary Figure 1: Quantifications for western blots presented in panels A (H3K9/14Ac and Acetyl lysine), C and D of figure 1. Signal quantifications from representative western blots, relative to total H3 immunoblot signals, are shown.

Supplementary Figure 2: Effects of NaBut and β-hydroxybutyrate molecules on histone acetylation in multiple cell types. (A) HEK293 cells were incubated for 18 hours with 5 mM NaBut, increasing concentrations of NaR-βOHB or TSA. Acid-extracted histones were immunoblotted with antibodies anti-H3K9/14Ac, anti total H3 and anti acetylated H2A.Z. **(B)** Primary human myotubes were incubated for 18 hours with 5 mM NaBut, 5 mM 4-PBA or the indicated hydroxybutyrates. Acid-extracted histones were immunoblotted with antibodies anti-H3K9/14Ac, anti acetylated H2A.Z or anti total histone H3 as loading control. Signal quantifications from representative western blots, relative to total H3 immunoblot signals, are shown on the right.

Chriett et al., supplementary Figure 1

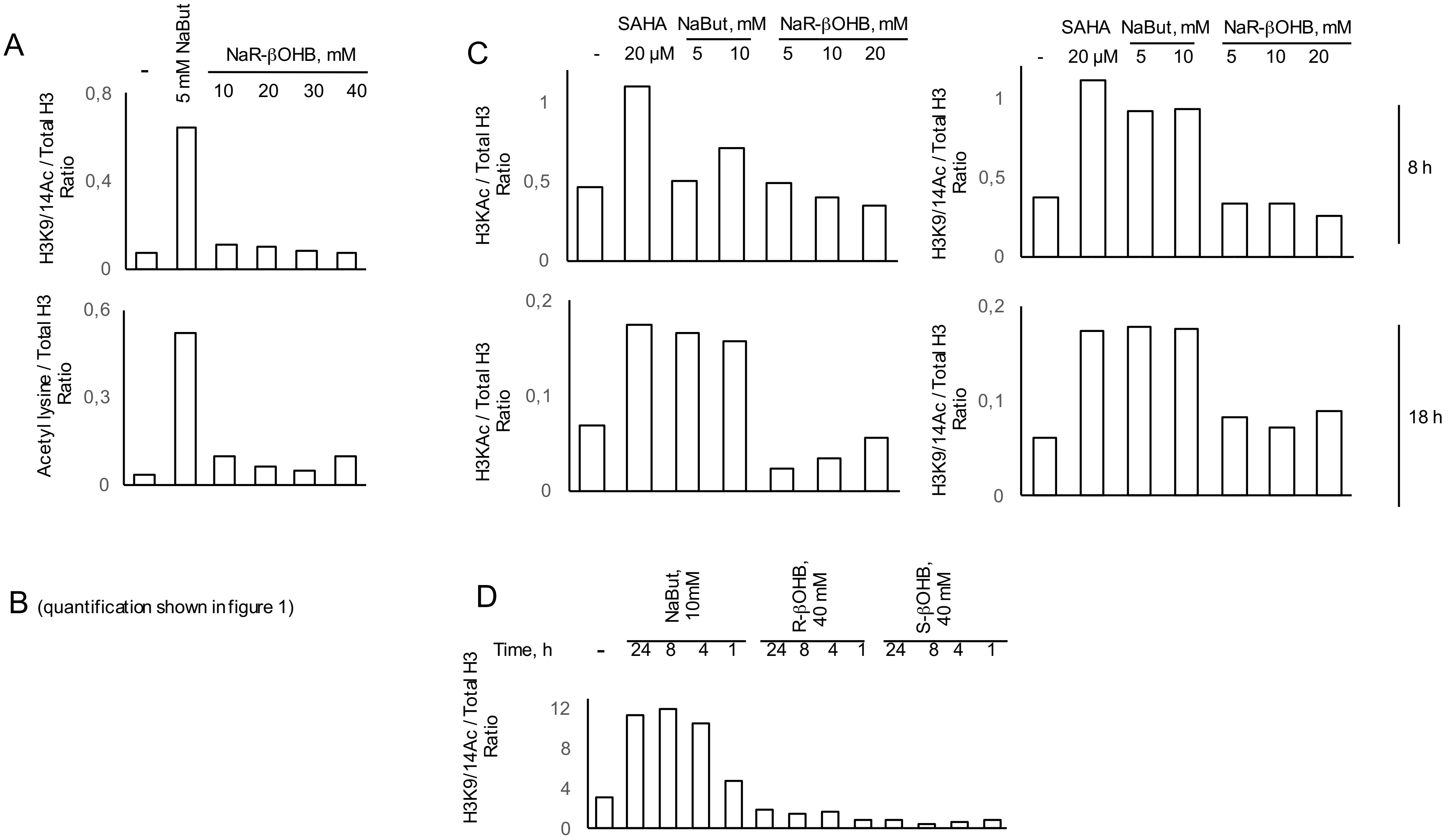

Chriett et al., Supplementary Figure 2

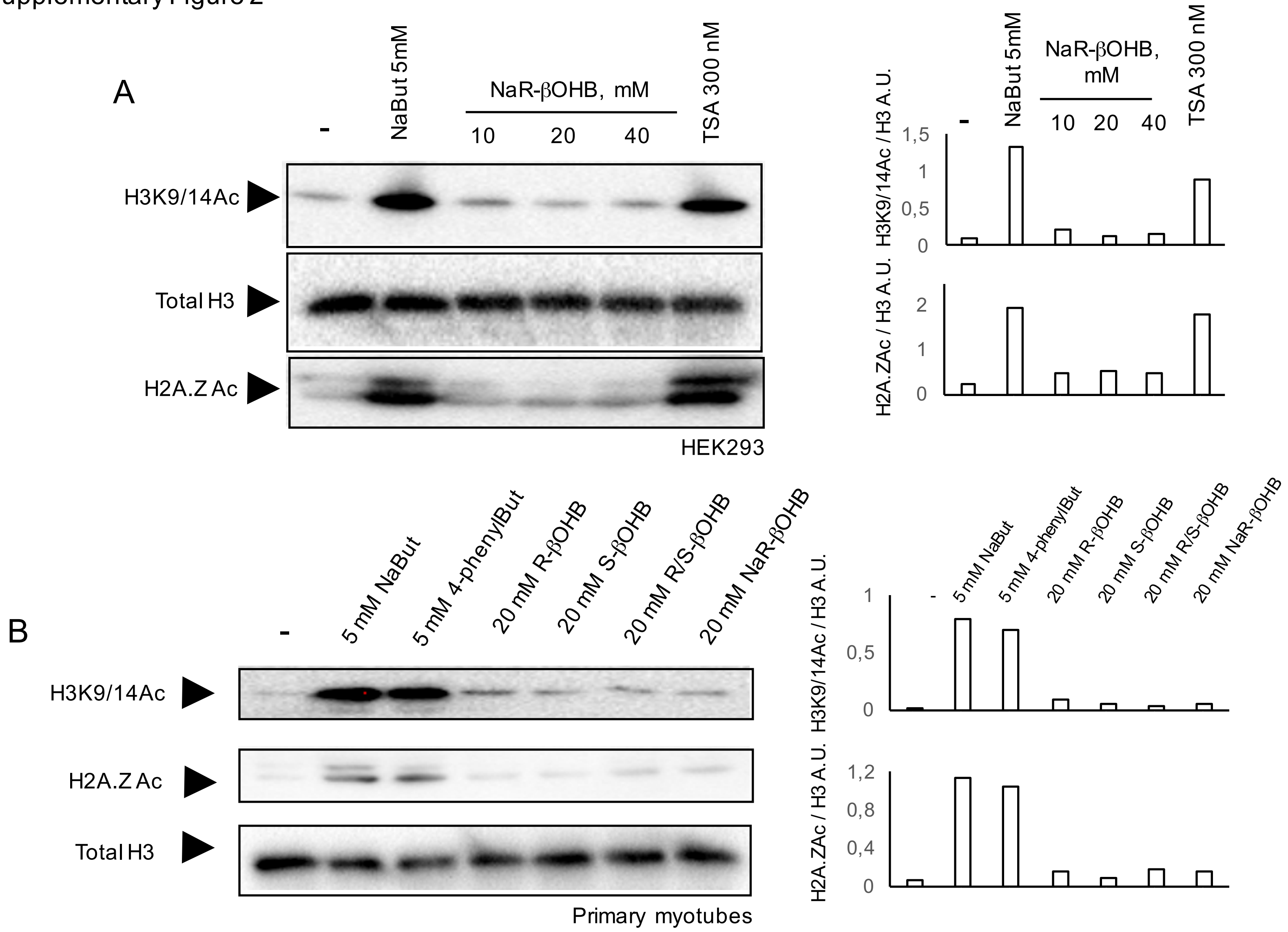

Figures with relative original data acquisition

Chriett et al., Figure 1 as submitted

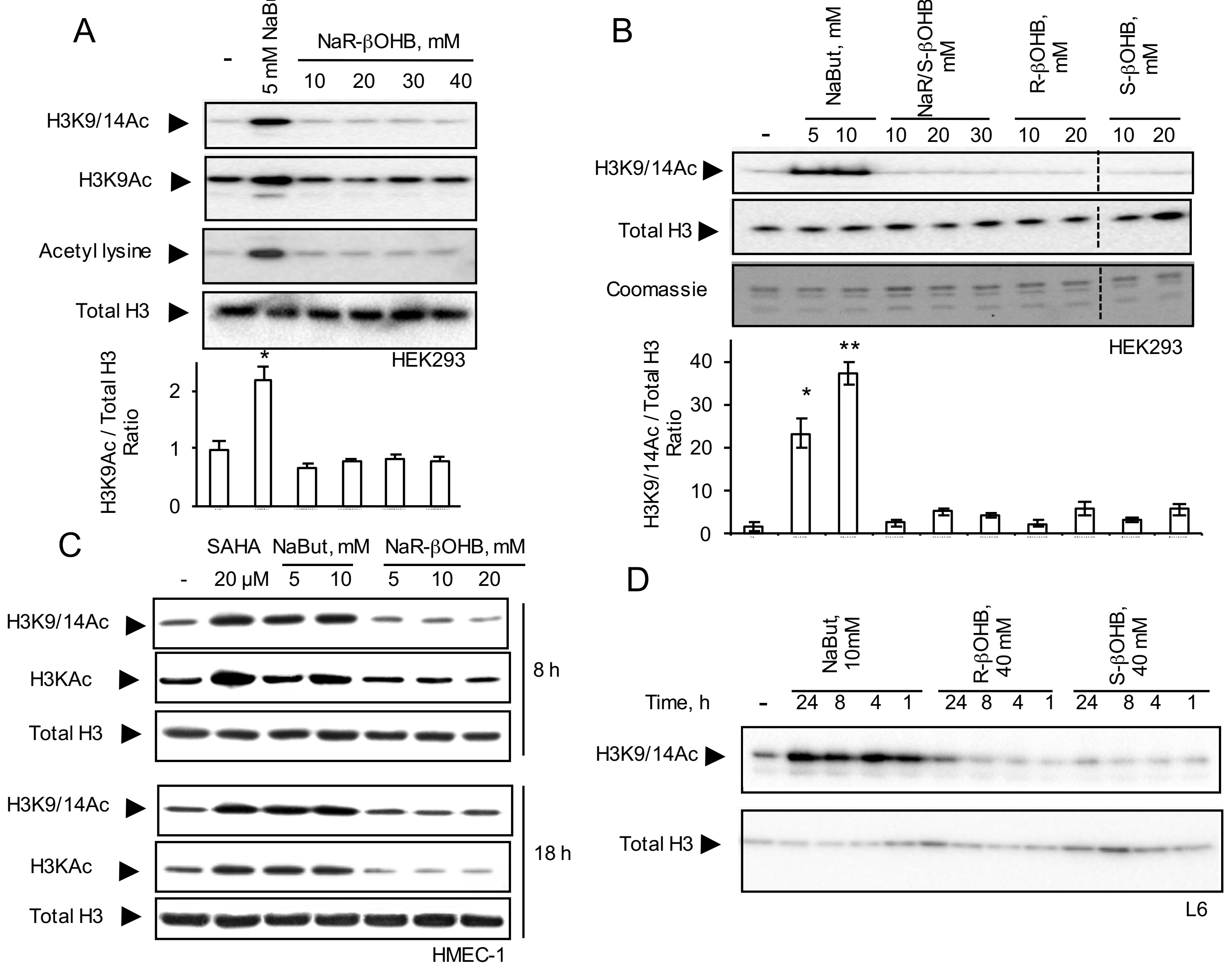

Chriett et al., Figure 1
Original scans of figure 1A

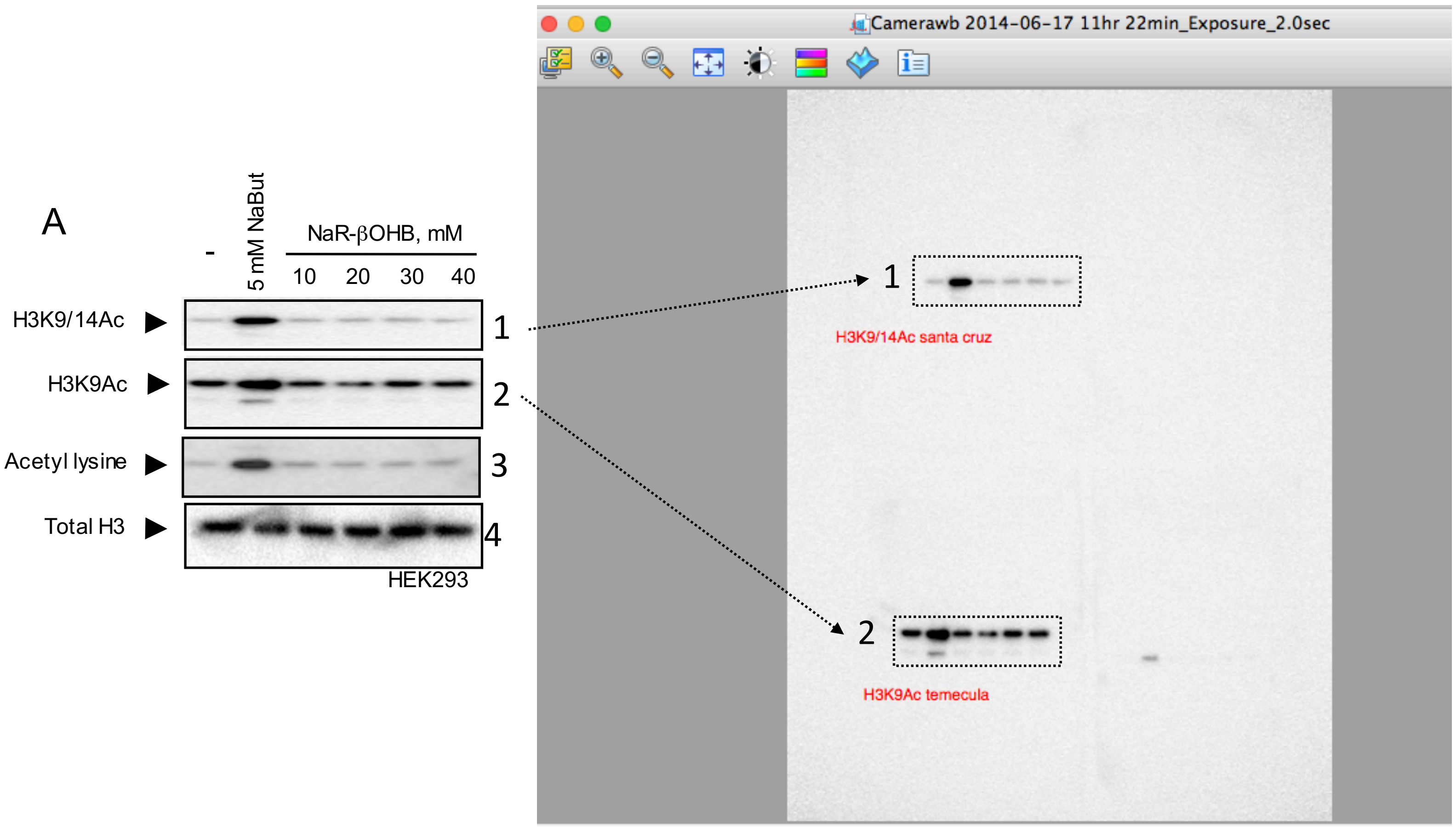

Blots acquired on a Biorad Chemidoc. Imagelab original file available.

Chriett et al., Figure 1
Original scans of figure 1A

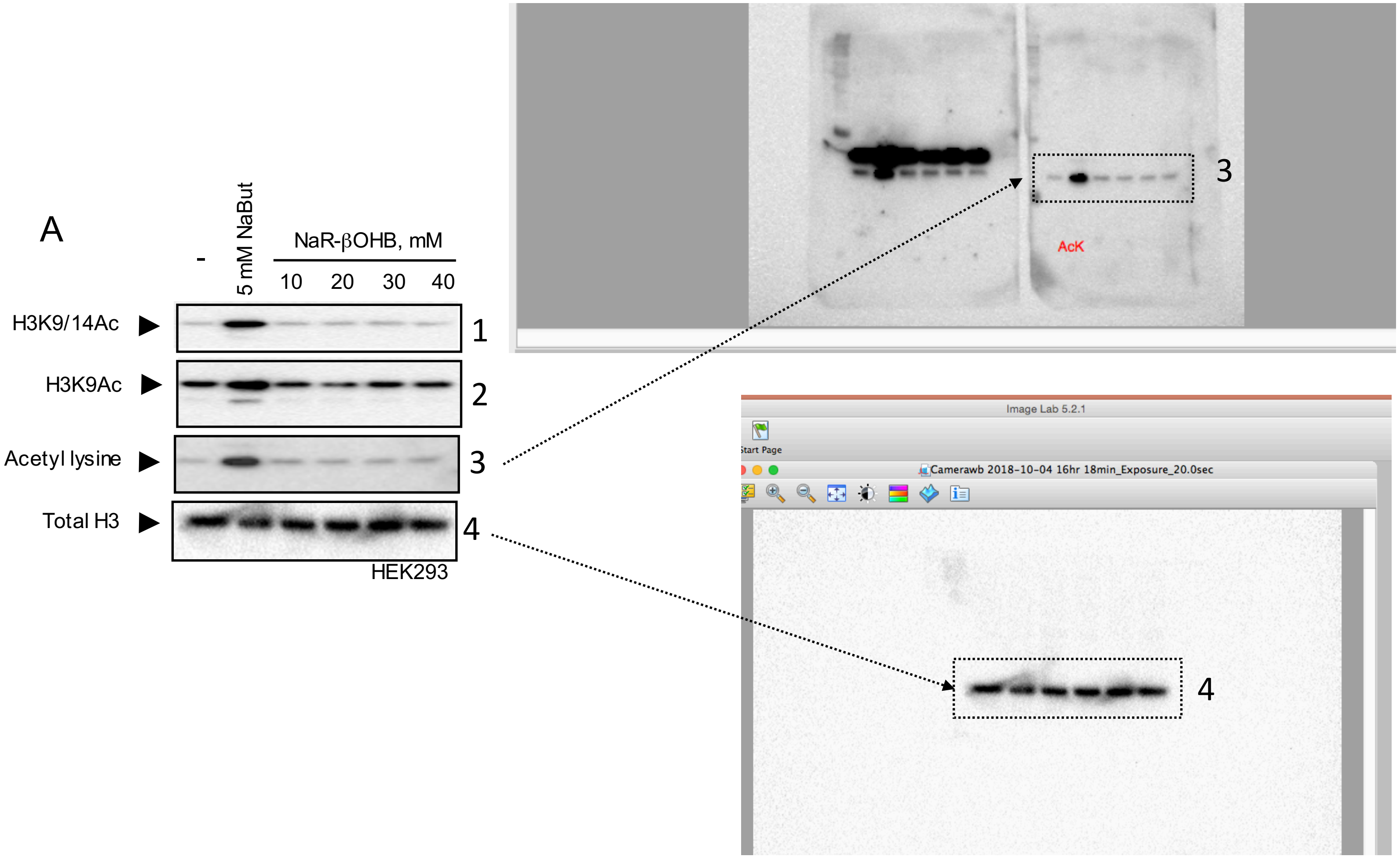

Blots acquired on a Biorad Chemidoc. Imagelab original file available.

Chriett et al., Figure 1
Original scans of figure 1B

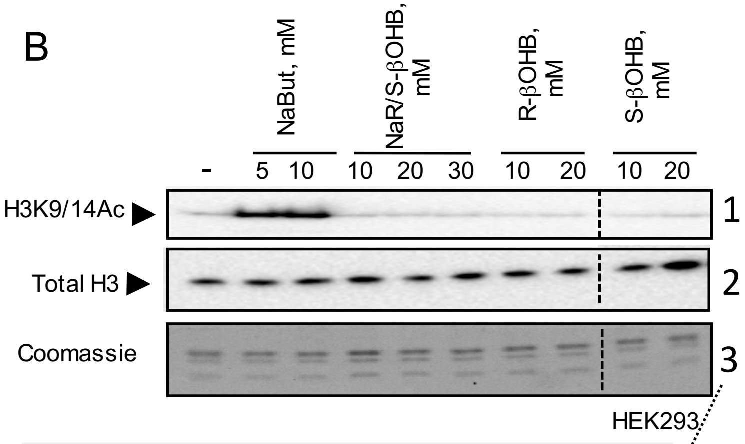

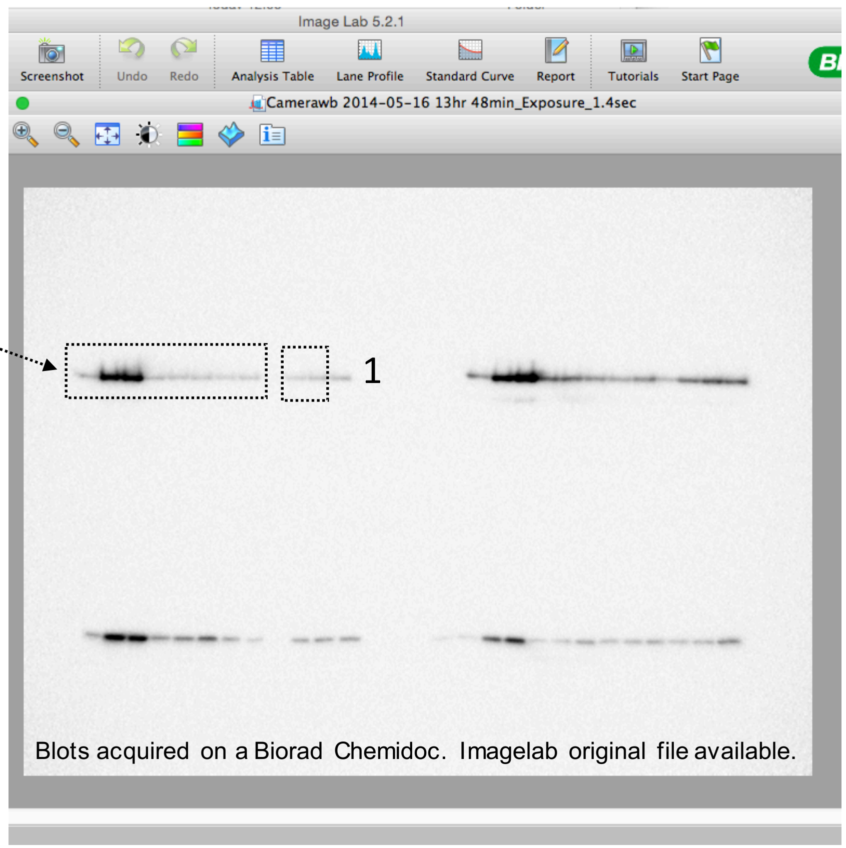

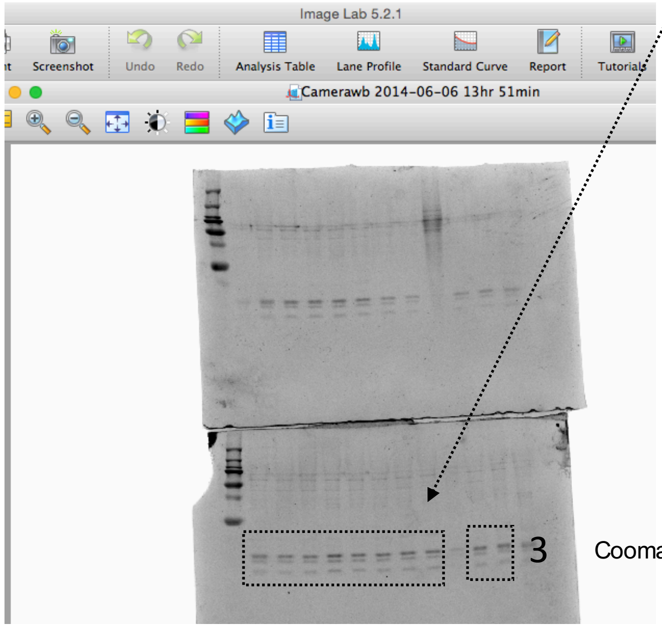

Coomassie blue photo acquired on a Biorad Chemidoc. Imagelab original file available.

Chriett et al., Figure 1
Original scans of figure 1B

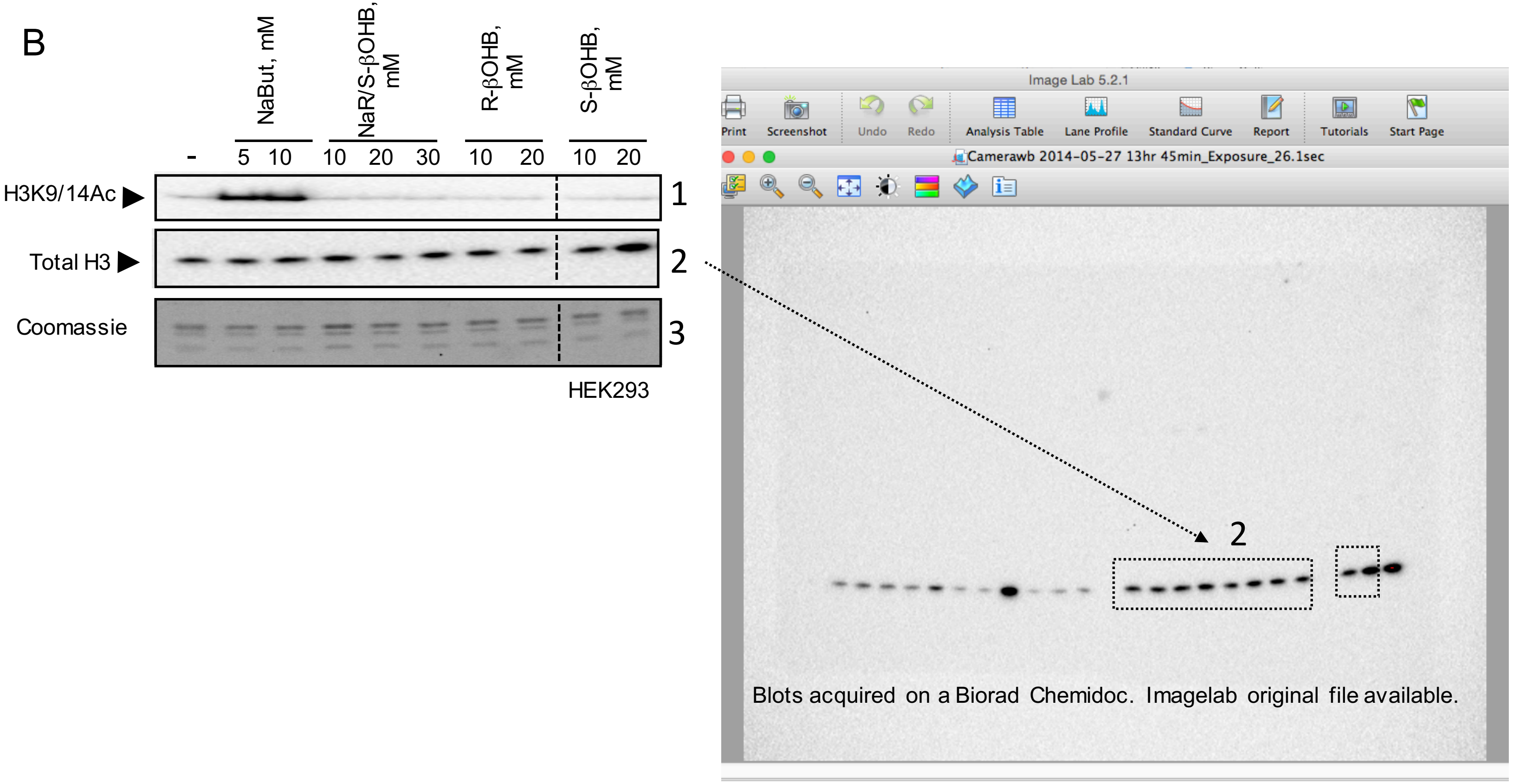

Chriett et al., Figure 1
Original scans of figure 1C

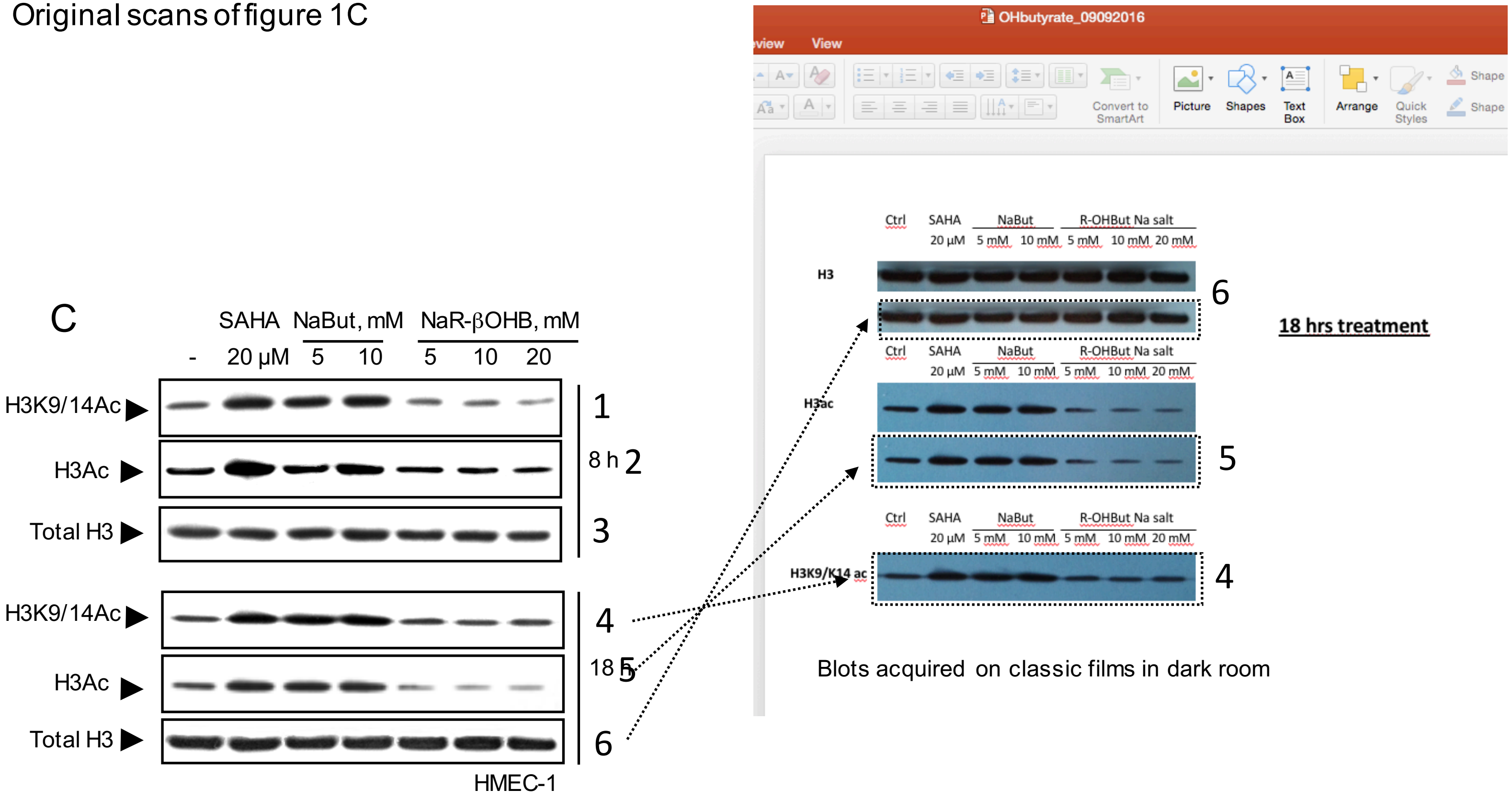

Chriett et al., Figure 1
Original scans of figure 1C

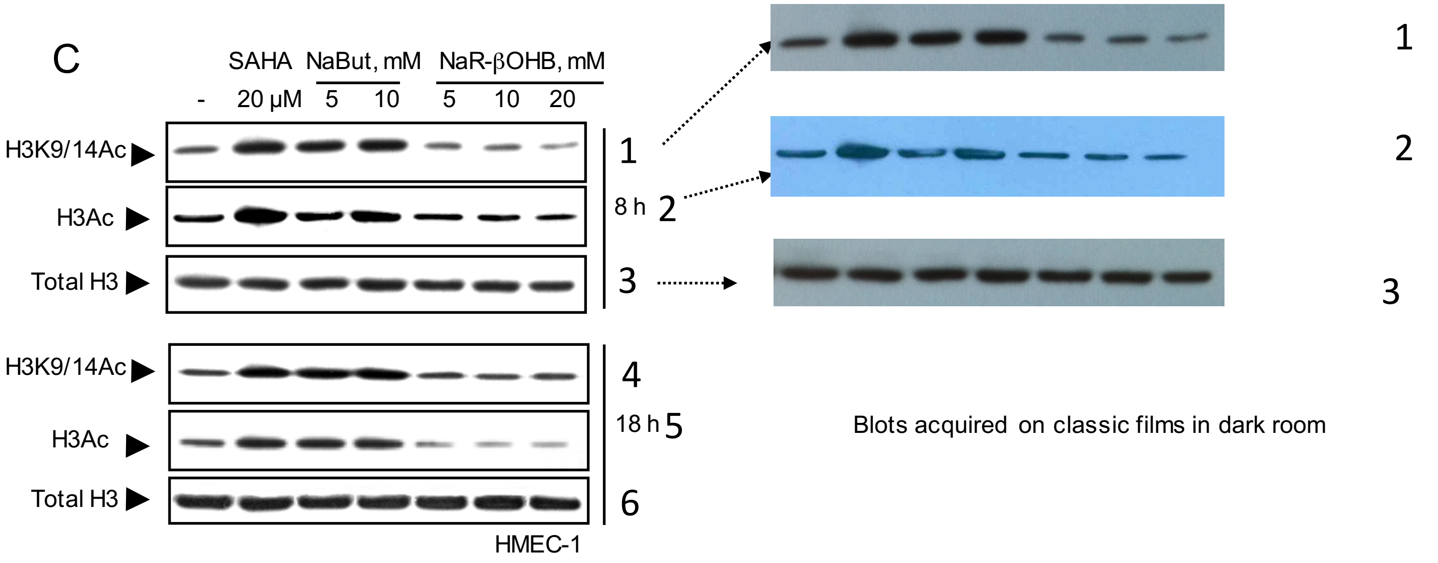

Chriett et al., Figure 1
Original scans of figure 1D

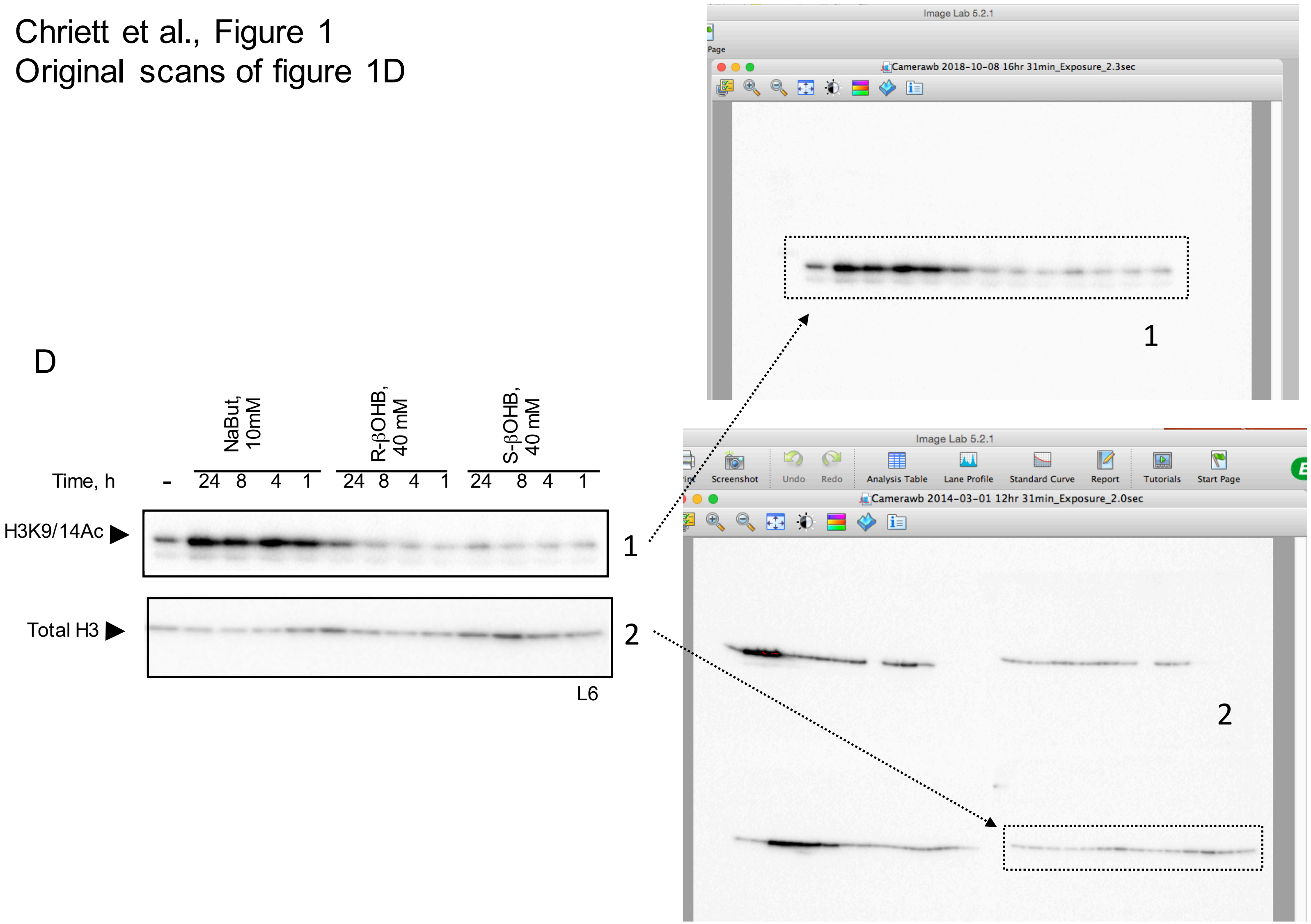

Blots acquired on a Biorad Chemidoc. Imagelab original file available.

Chriett et al., Figure 3 as submitted

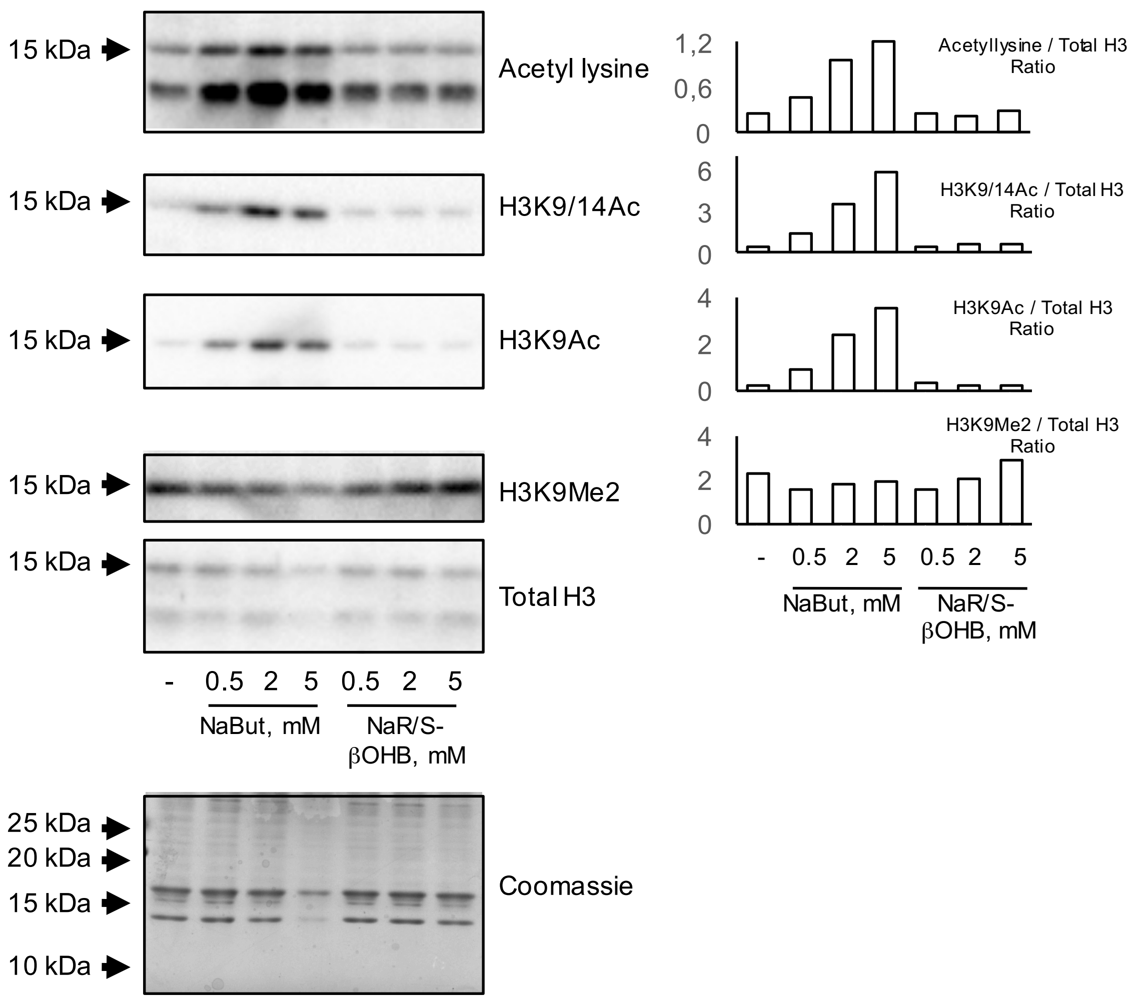

Chriett et al., Figure 3
Original scans of figure 3

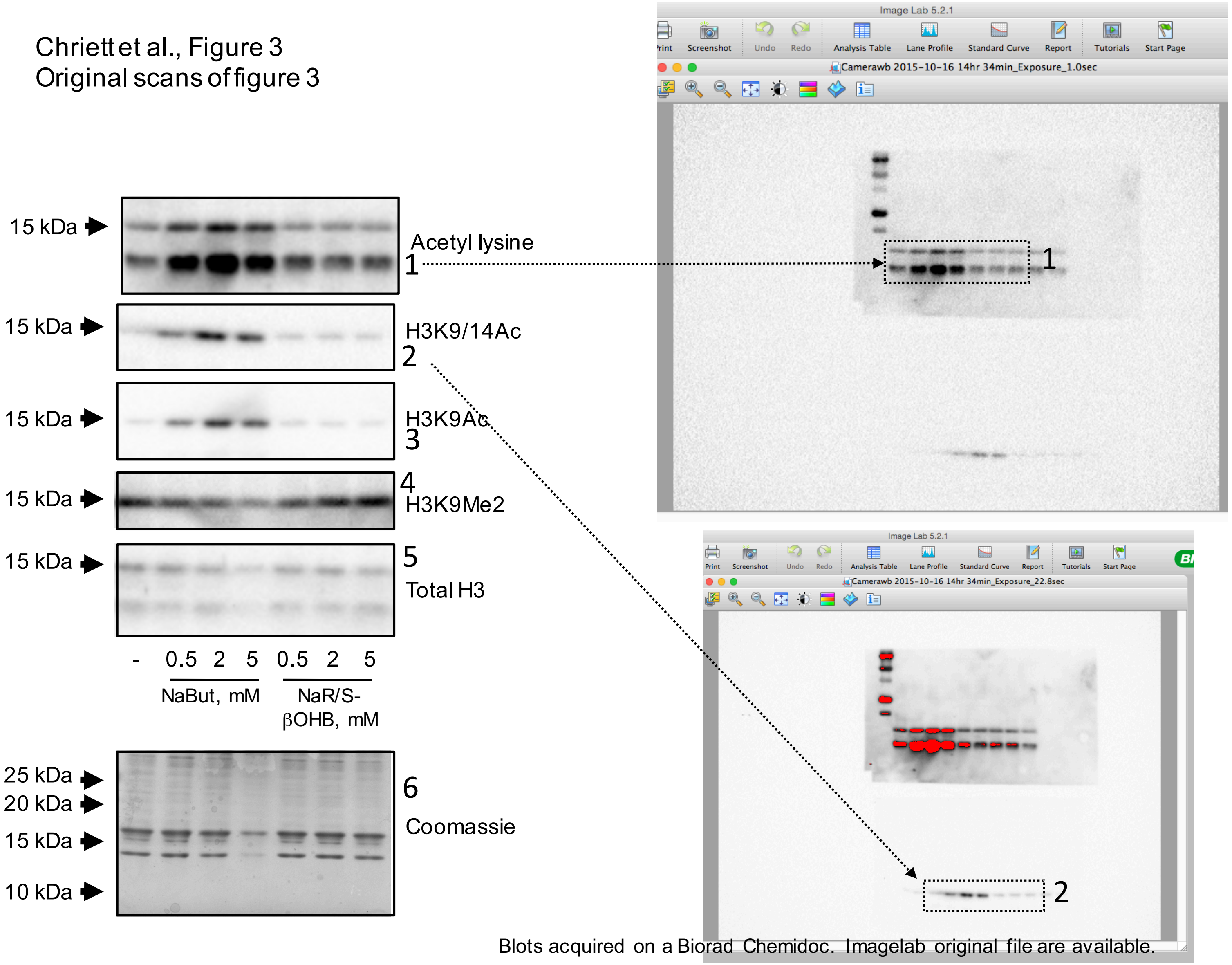

Blots acquired on a Biorad Chemidoc. Imagelab original file are available.

Chriett et al., Figure 3
Original scans of figure 3

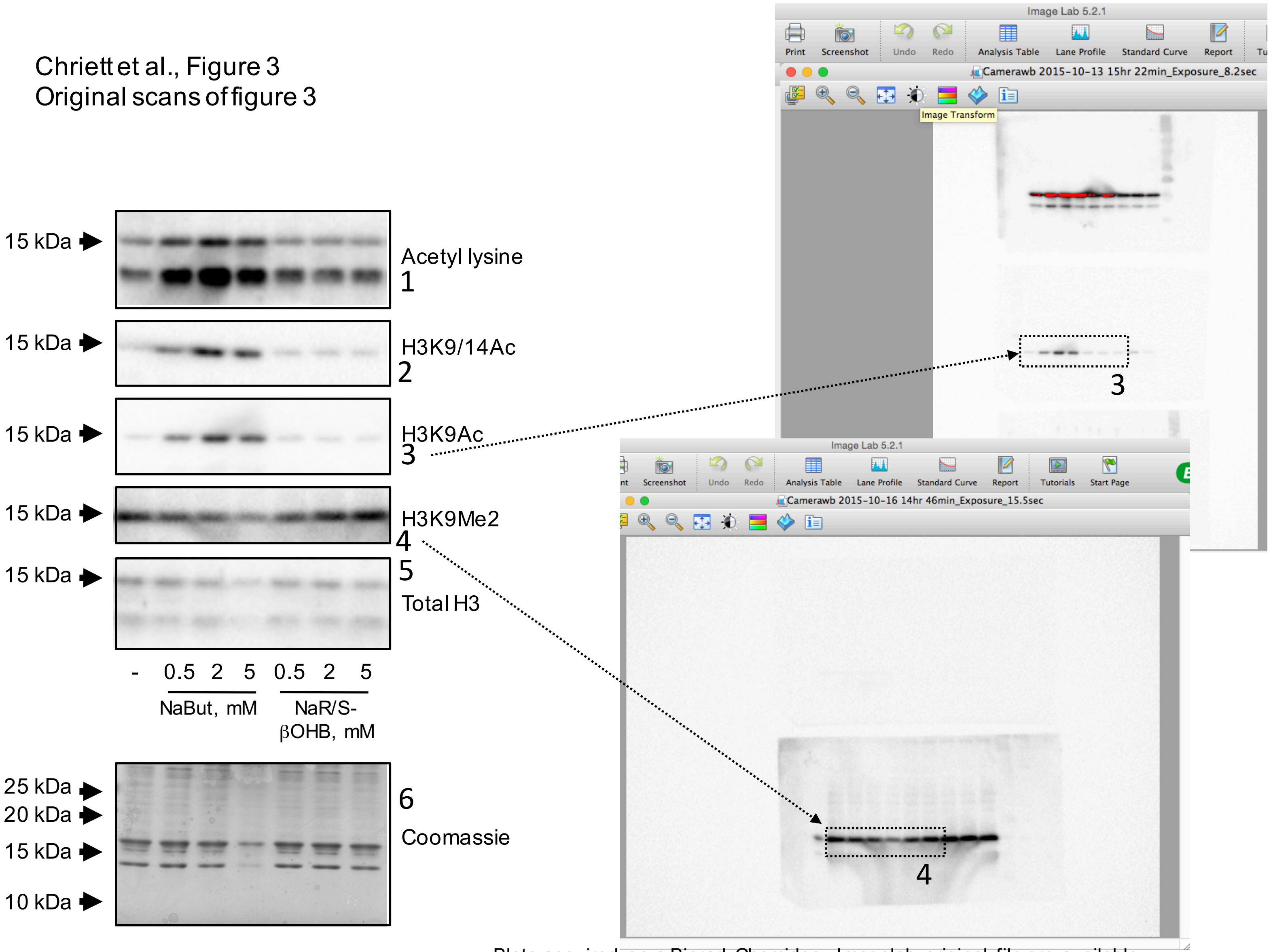

Blots acquired on a Biorad Chemidoc. Imagelab original file are available.

Chriett et al., Figure 3
Original scans of figure 3

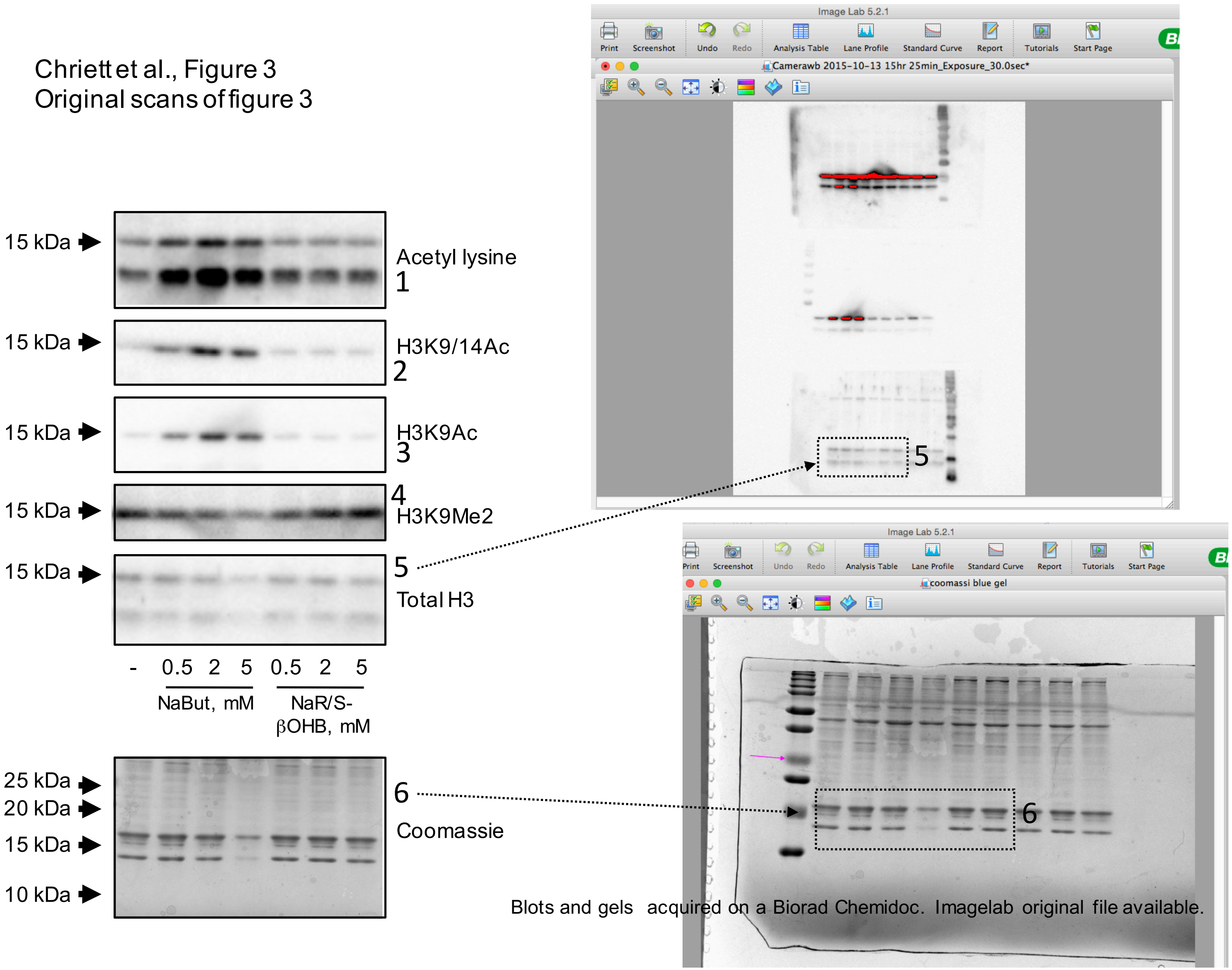

Blots and gels acquired on a Biorad Chemidoc. Imagelab original file available.

Chriett et al., Figure 4 as submitted

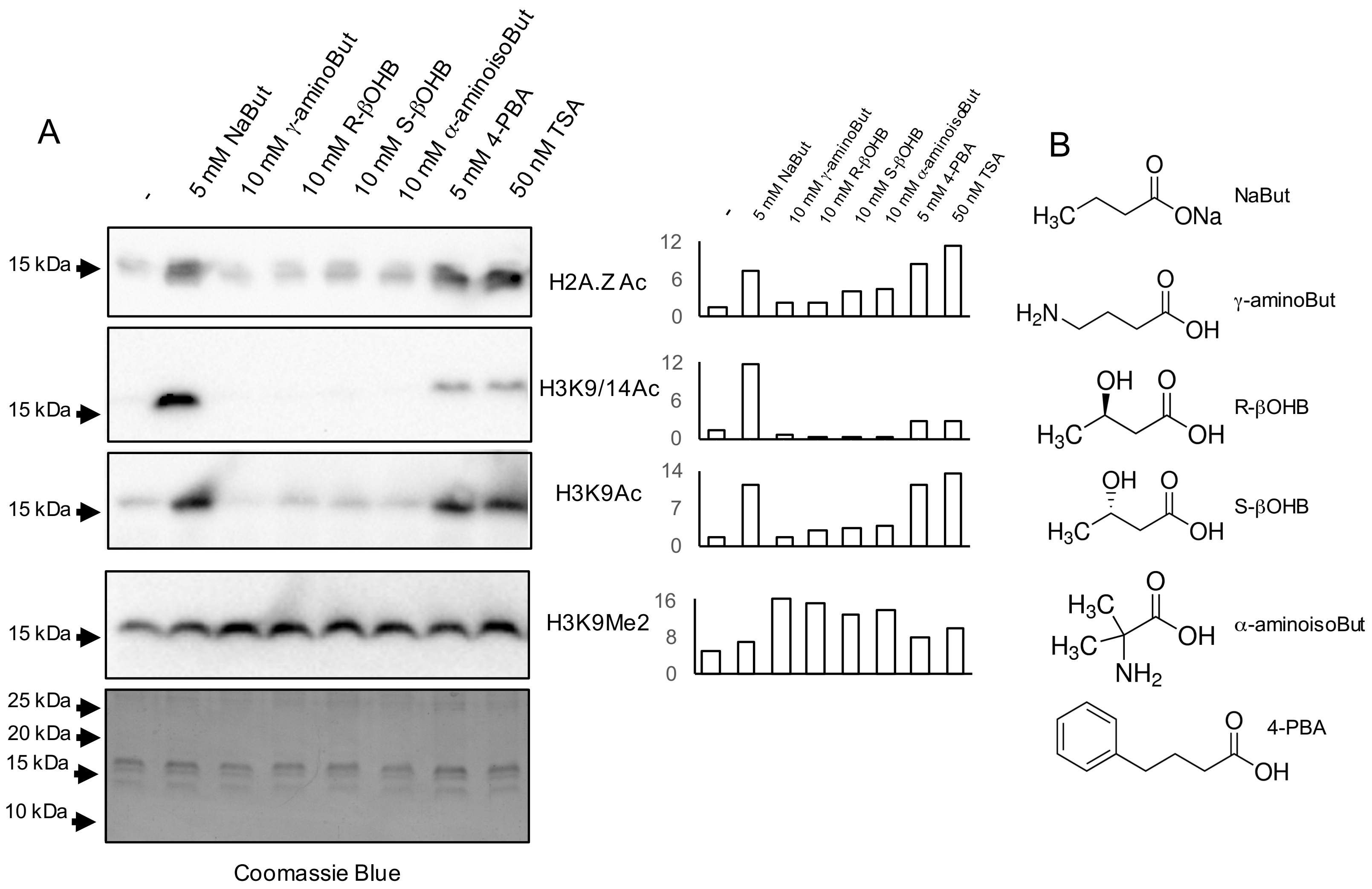

Chriett et al., Figure 4
Original scans

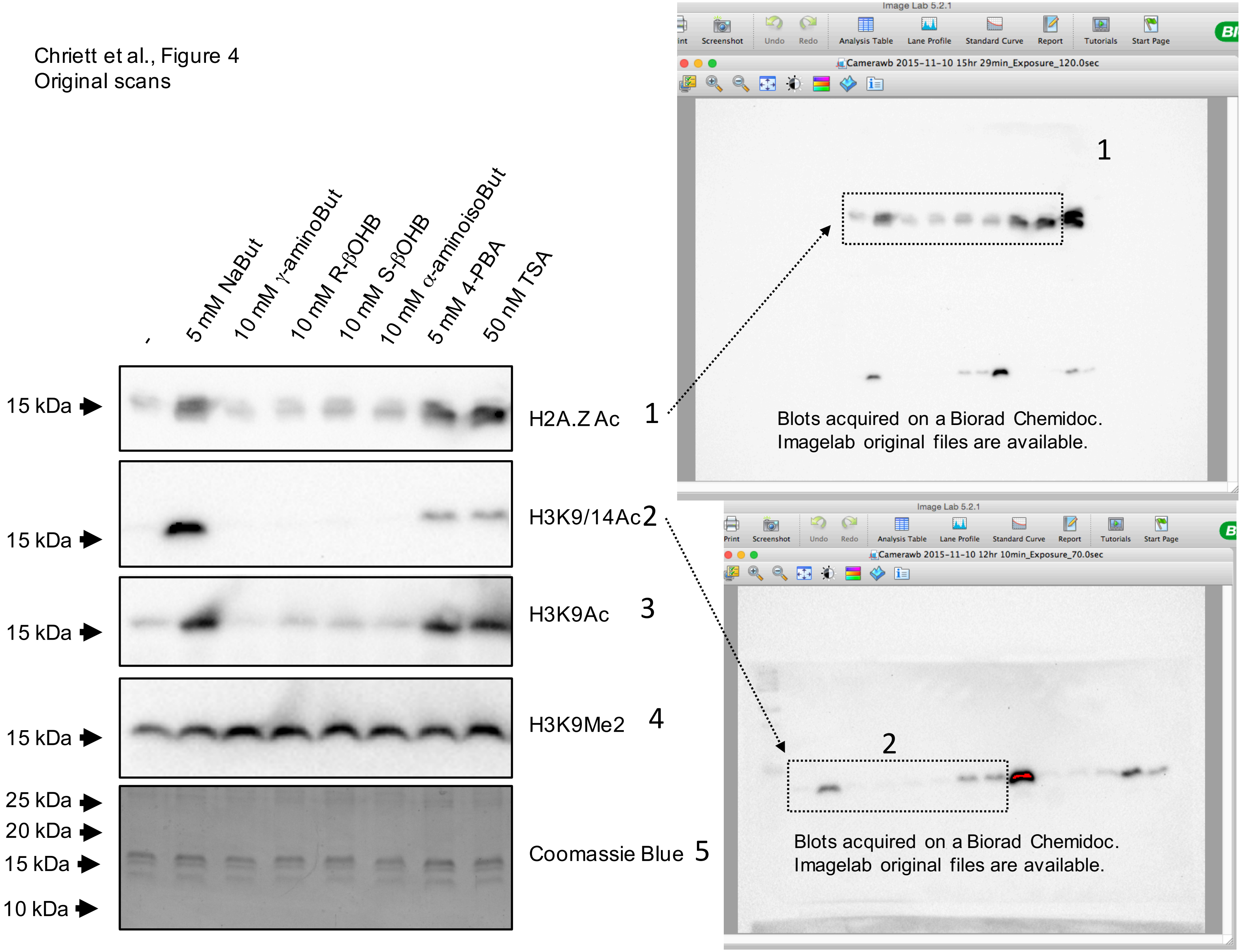

Chriett et al., Figure 4
Original scans

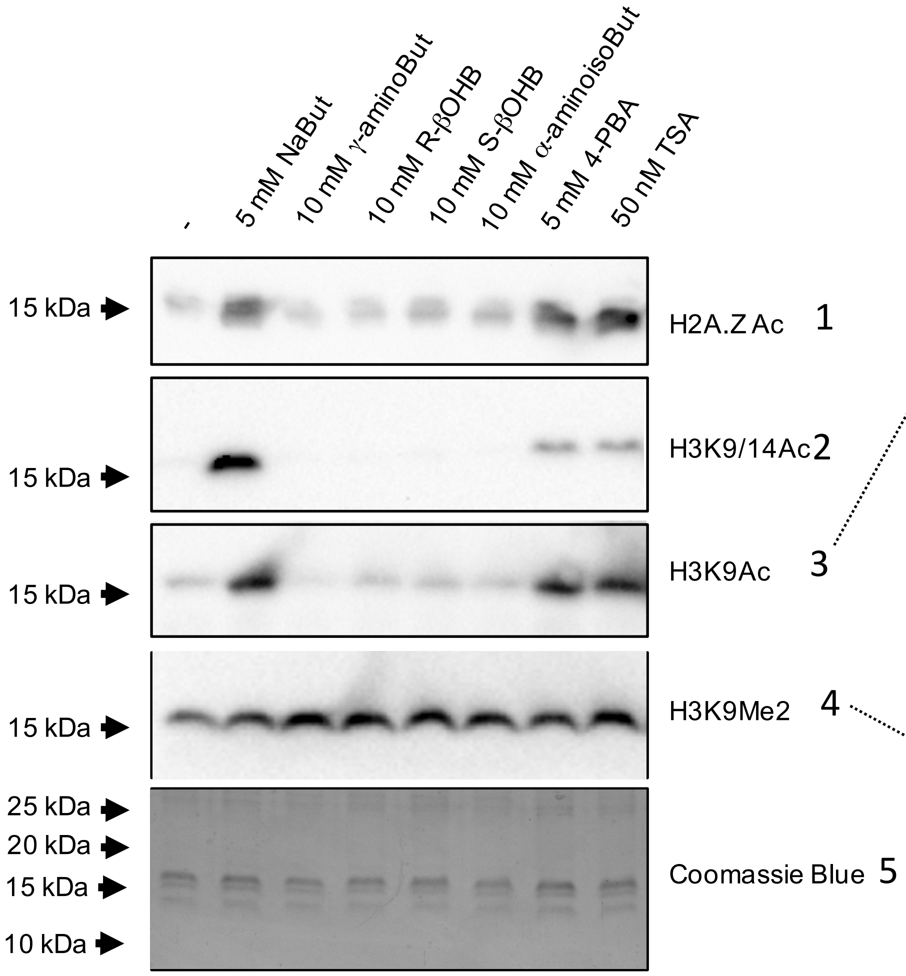

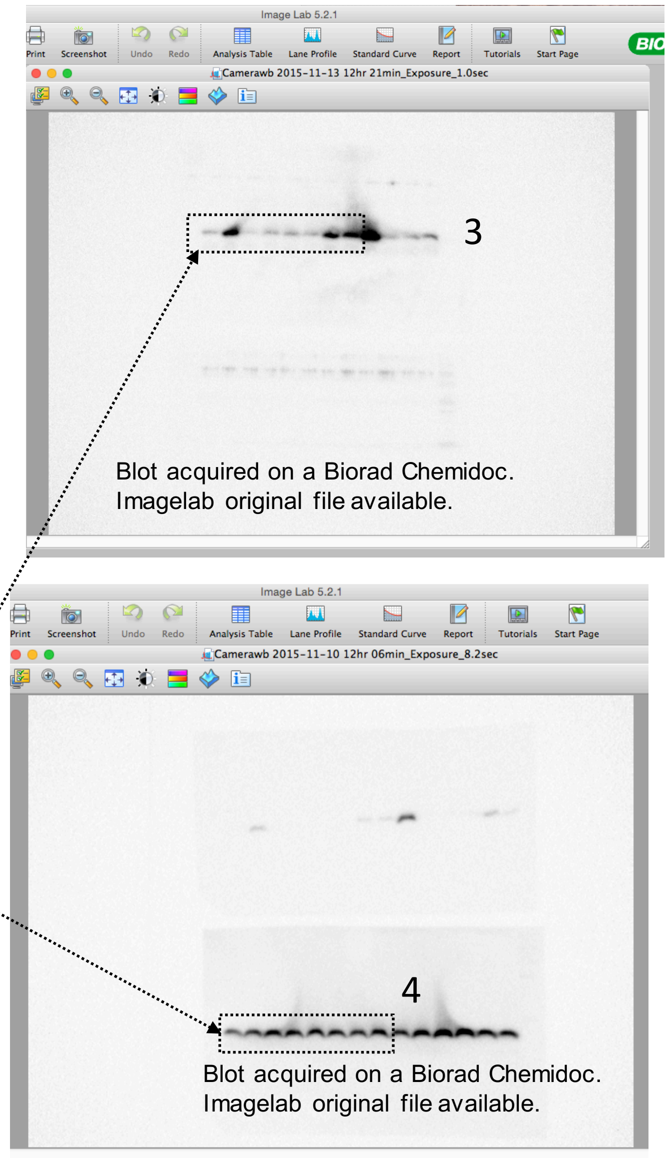

Chriett et al., Figure 4
Original scans

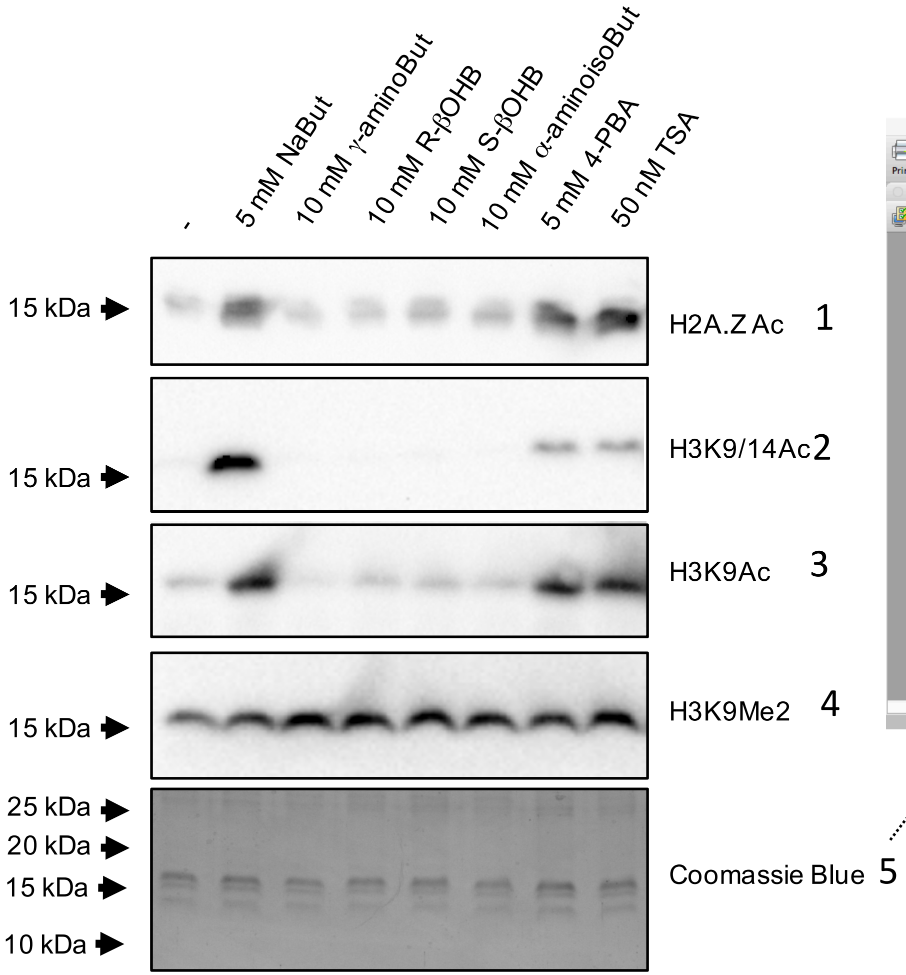

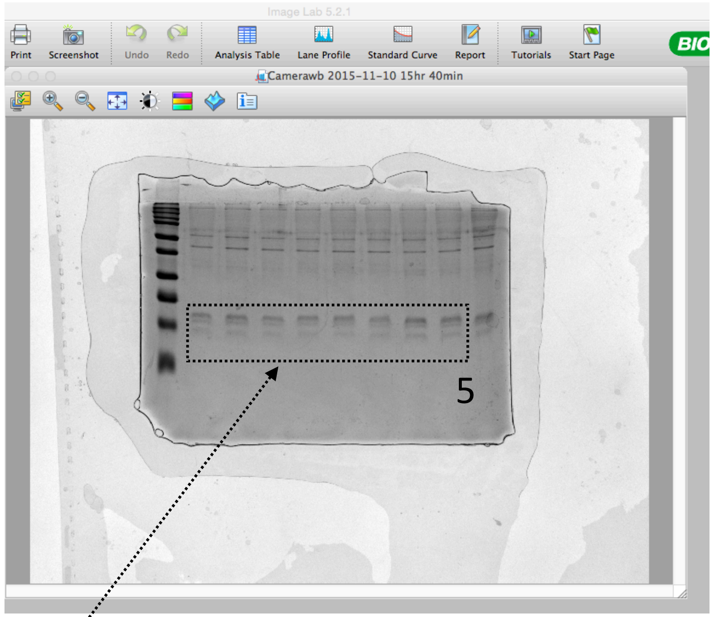

Coomassie blue photo acquired on a Biorad Chemidoc. Imagelab original file available.

Chriett et al., Figure 5 as submitted

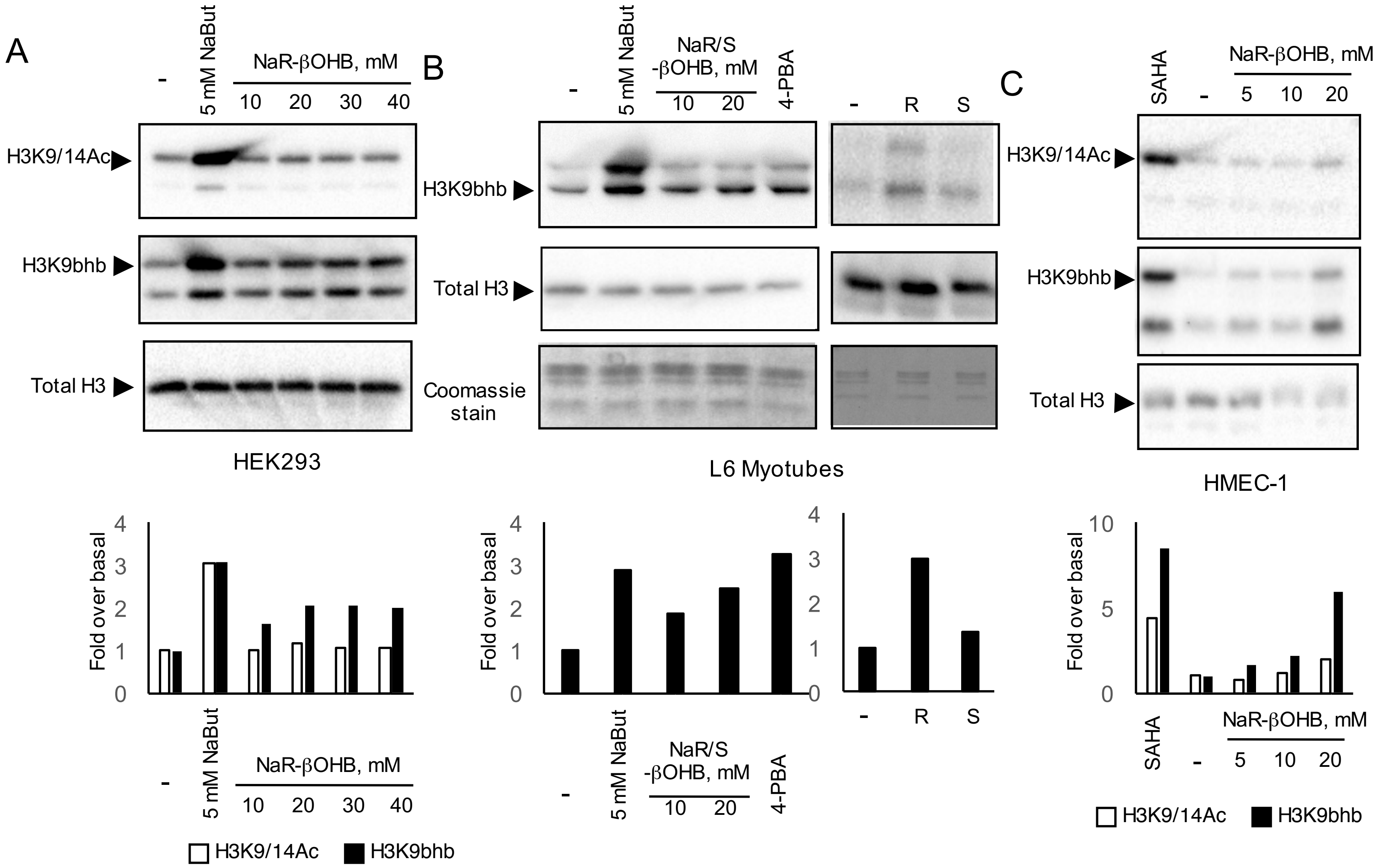

Chriett et al., Figure 5
Original scans of figure 5A

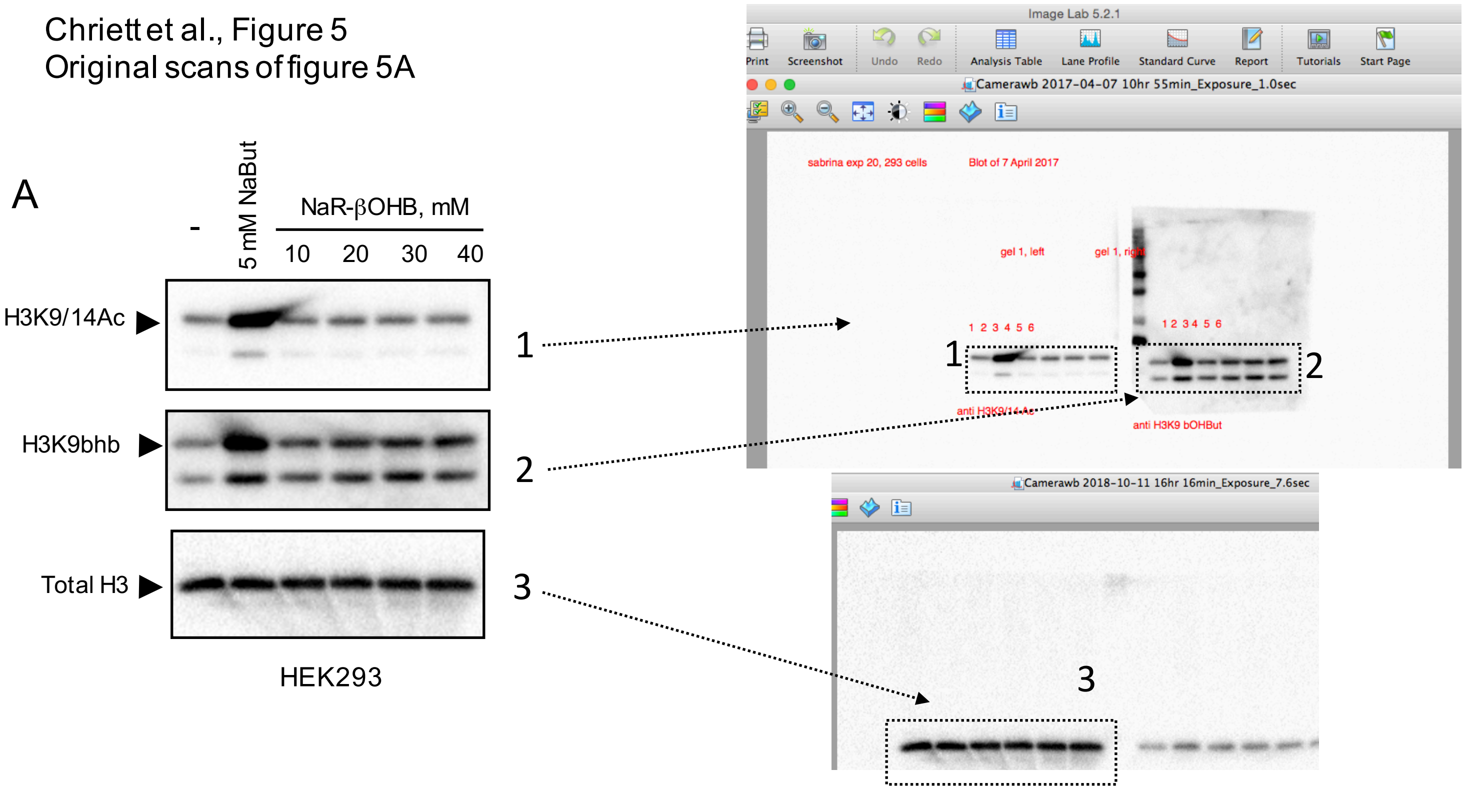

Blots acquired on a Biorad Chemidoc.
Imagelab original files are available.

Chriett et al., Figure 5
Original scans of figure 5B

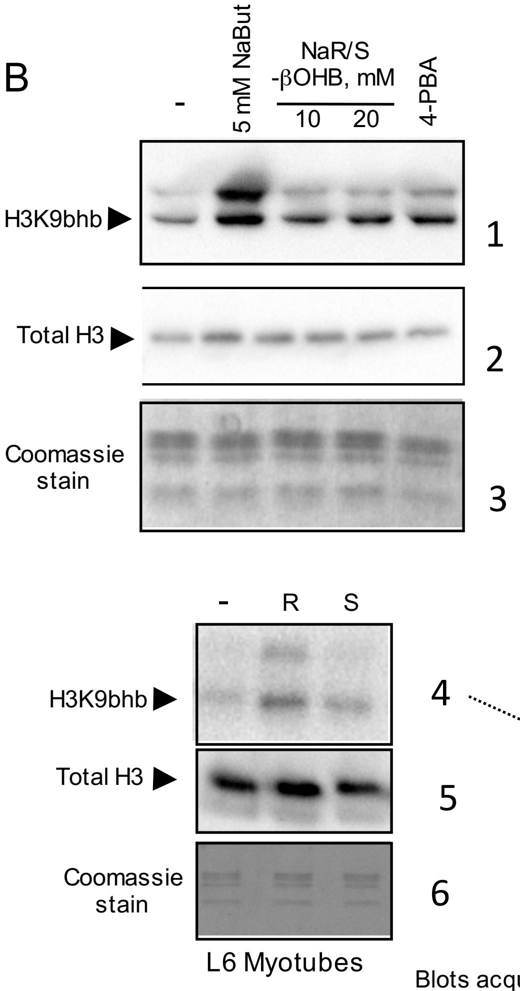

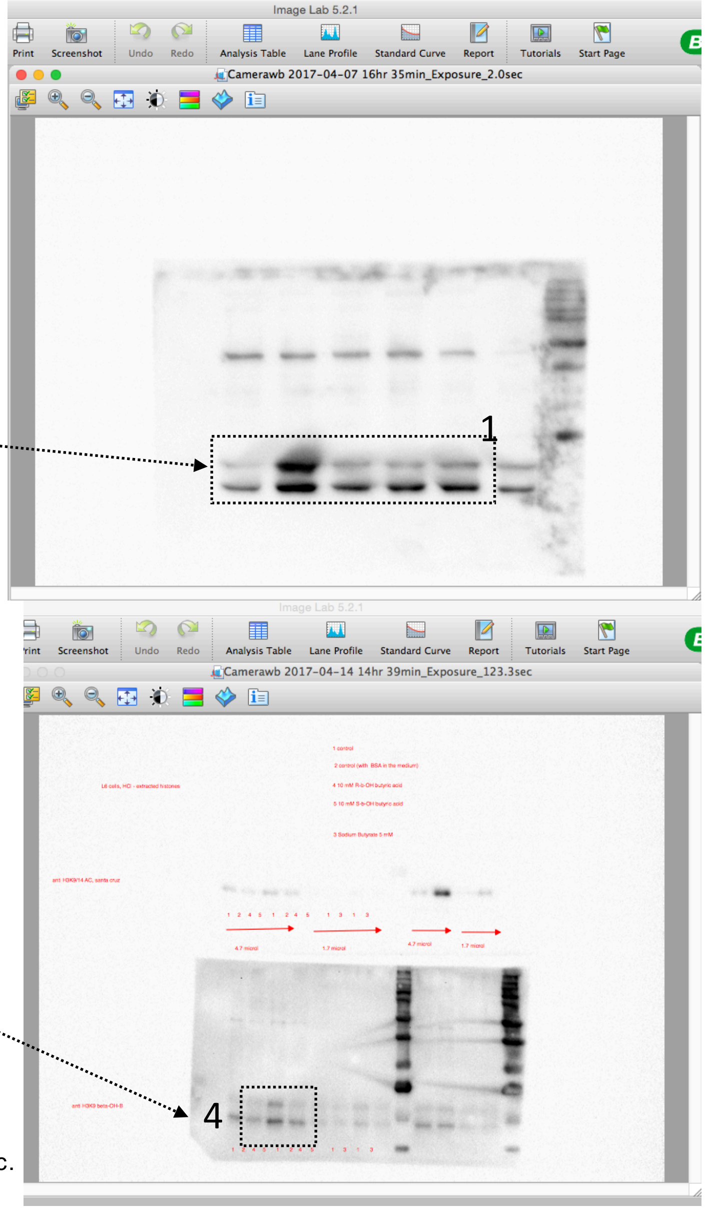

Blots acquired on a Biorad Chemidoc.
Imagelab original files are available.

Chriett et al., Figure 5
Original scans of figure 5B

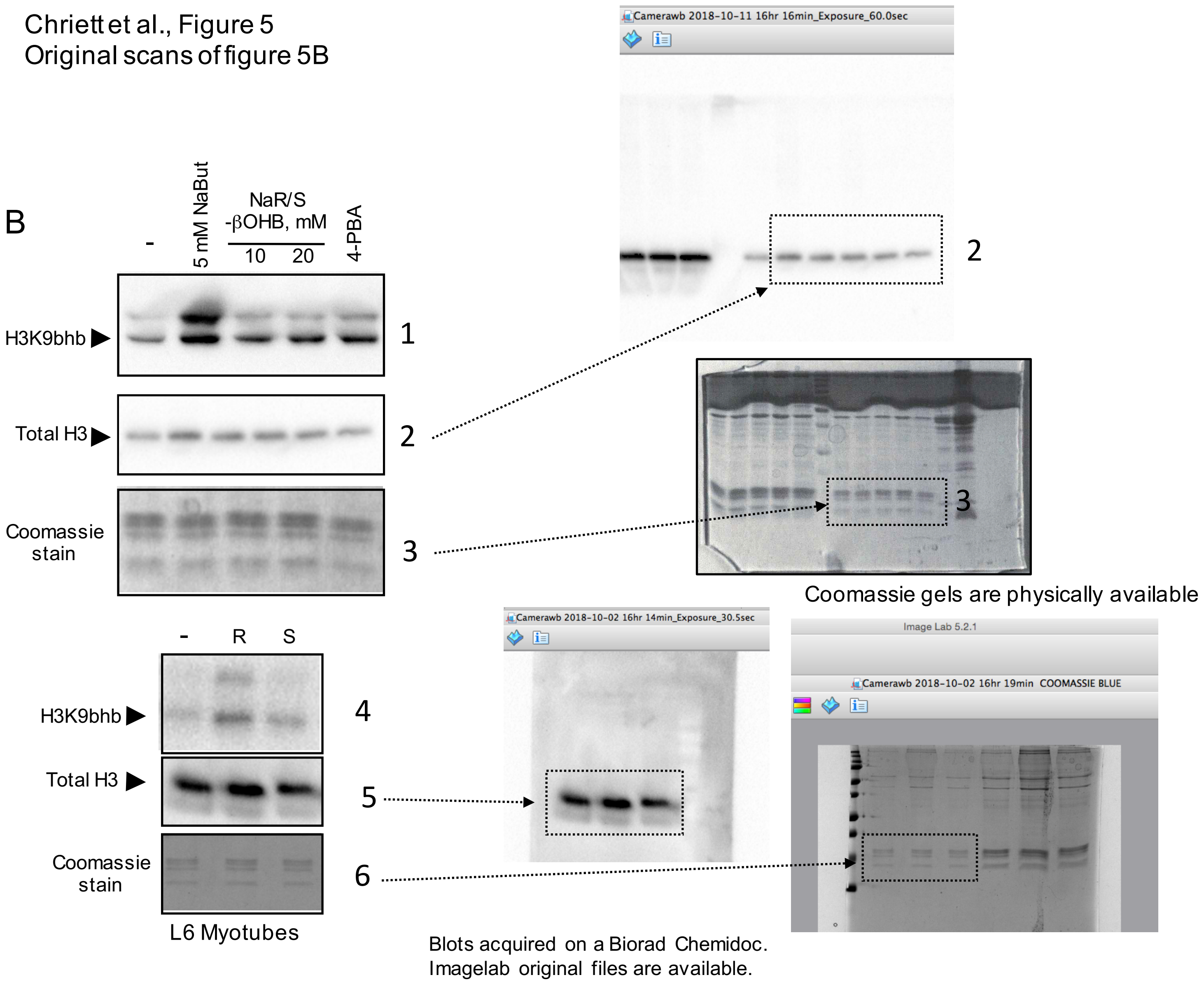

Chriett et al., Figure 5
Original scans of figure 5C

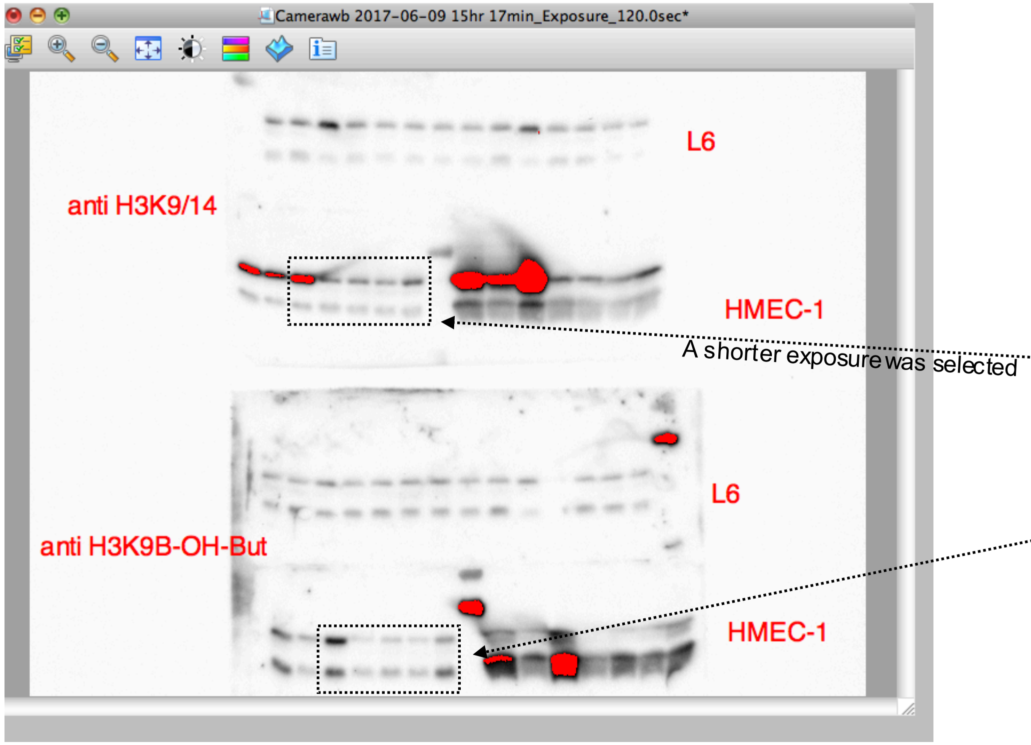

Blots acquired on a Biorad Chemidoc.
Imagelab original files are available.

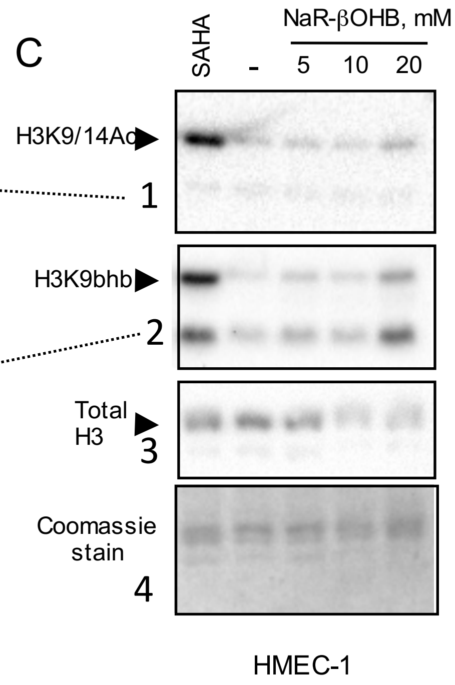

Chriett et al., Figure 5
Original scans of figure 5C

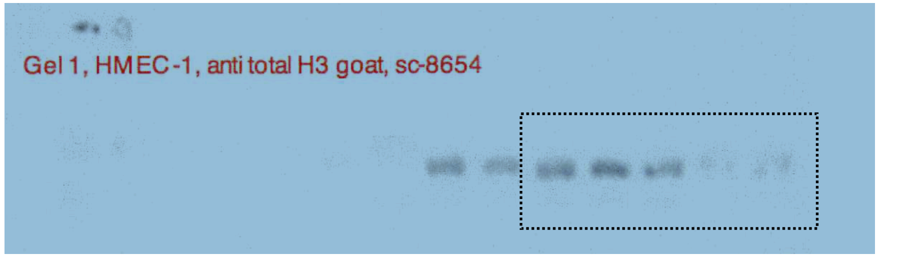

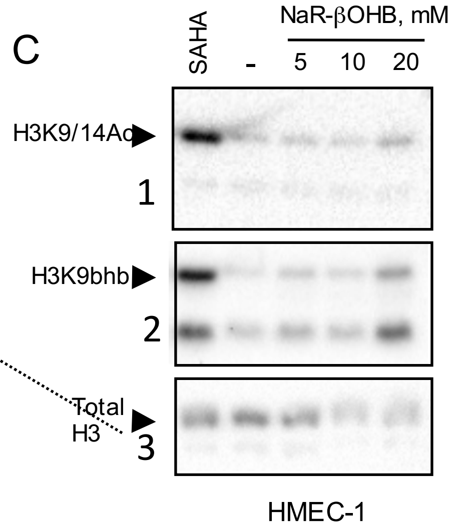

Chriett et al., figure 10 as submitted

A

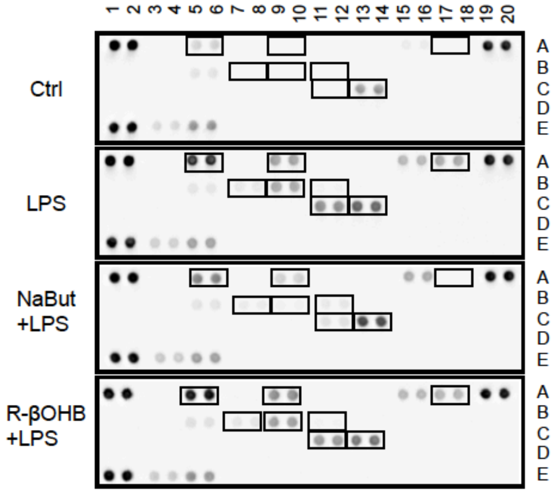

1, 2	3, 4	5, 6	7, 8	9, 10	11, 12	13, 14	15, 16	17, 18	19, 20	
CTR(+)	C5/C5a	**CD40 Ligand**	G-CSF	**GM-CSF**	GR0α	I-309	sICAM-1	**INF-γ**	CTR(+)	A
-	IL-1α	IL-1β	**IL-1ra**	**IL-2**	**IL-4**	IL-5	IL-6	IL-8	-	B
-	IL-10	IL-12p70	IL-13	IL-16	**IL-17**	**IL-17E**	IL-23	IL-27	-	C
-	IL-32α	IP-10	I-TAC	MCP-1	MIF	MIP-1α	MIP-1β	Serpin E1	-	D
CTR(+)	RANTES	SDF-1	TNF-α	sTREM-1	-	-	-	-	CTR(-)	E

B

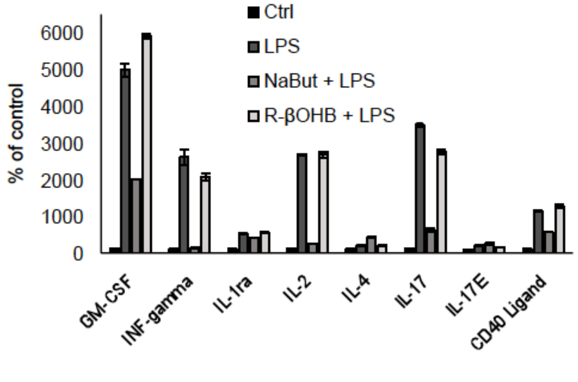

Chriett et al., Figure 10

Original scan of figure 10

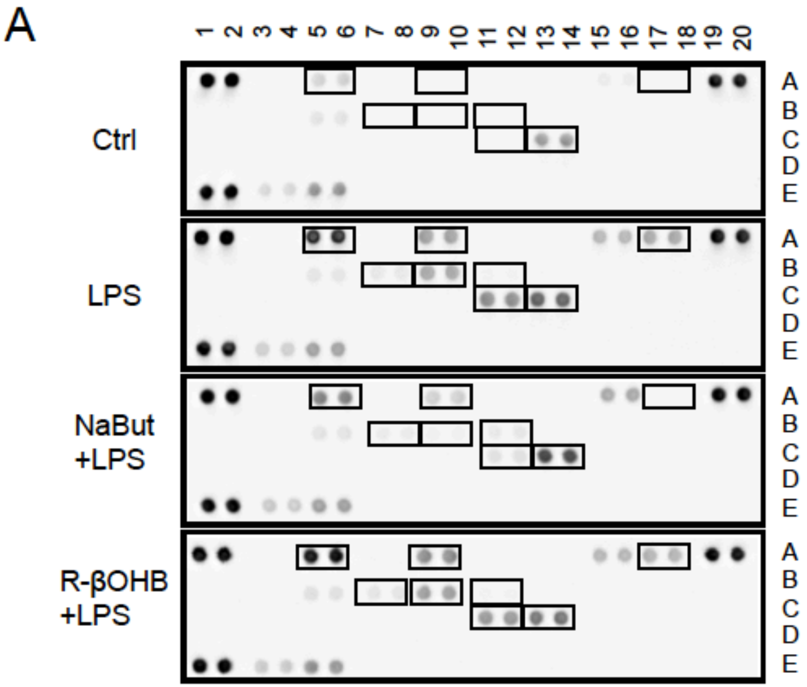

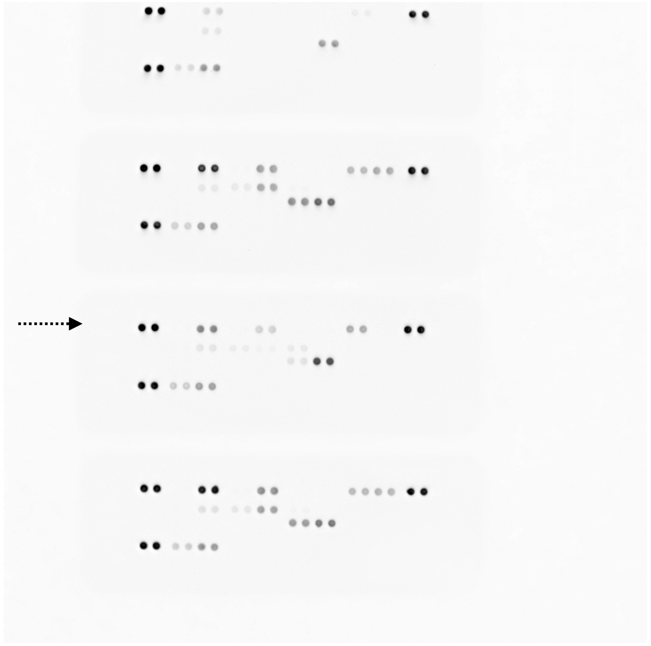

Original dot blots (membranes were visualised using CCD digital imaging system Alliance Mini HD4 (UVItec Limited, Cambridge, United Kingdom. Files of the original acquisition on the CCD digital imaging system are available).

Chriett et al., Supplementary Figure 2 as submitted

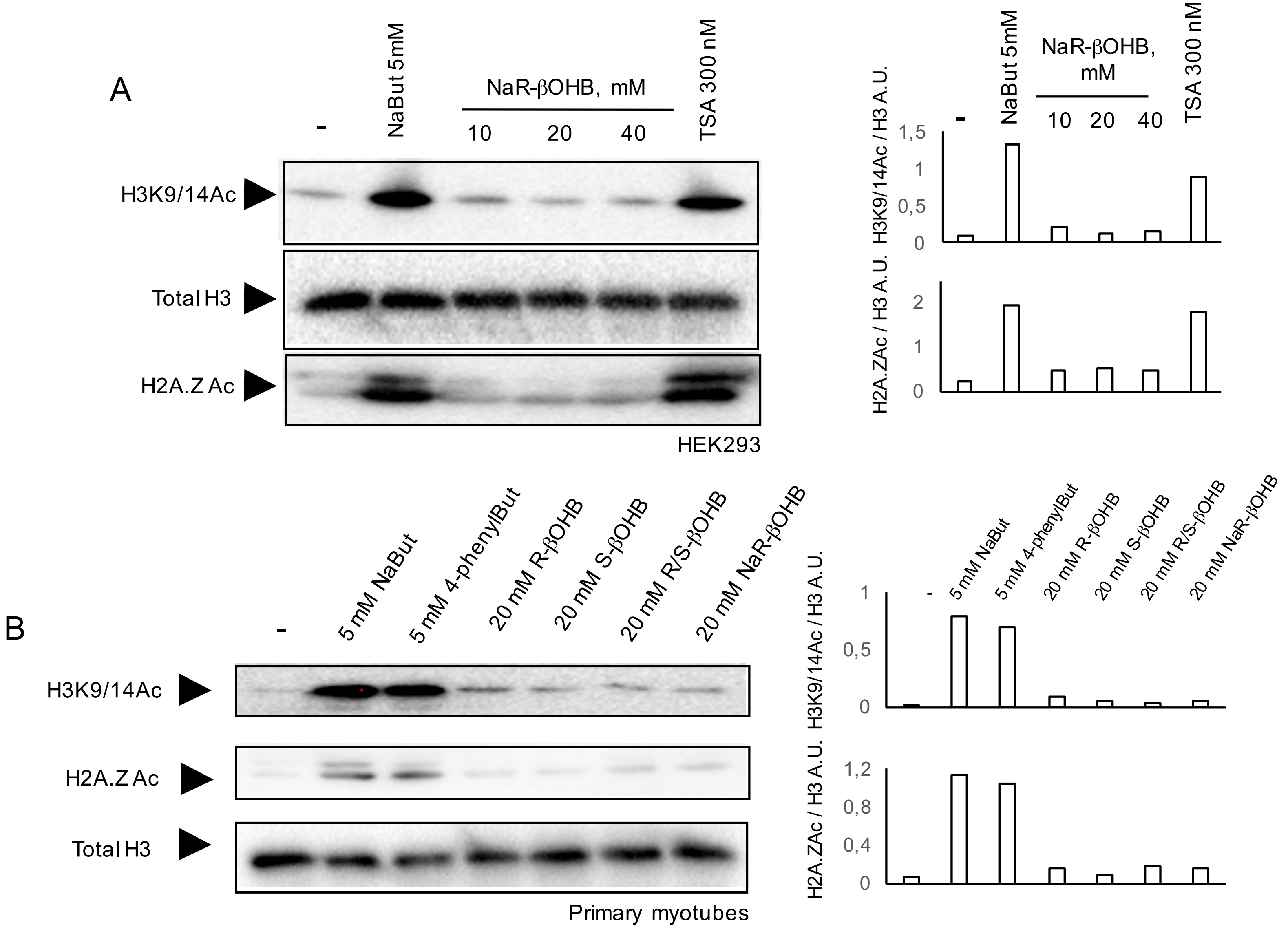

Chriett et al., Supplementary Figure 2
Original scans of Supplementery figure 2A

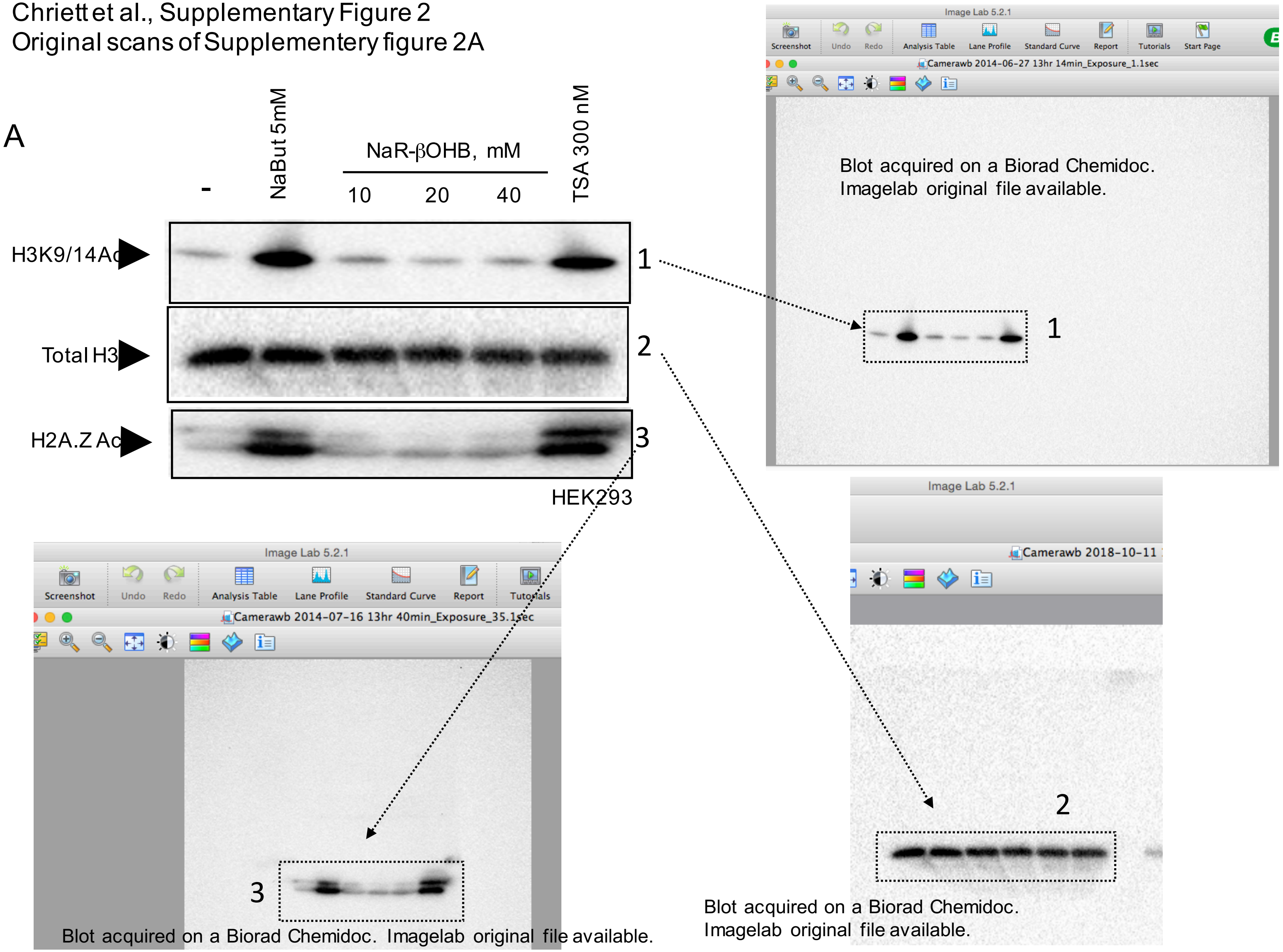

Chriett et al., Supplementary Figure 2
Original scans of supplementery figure 2B

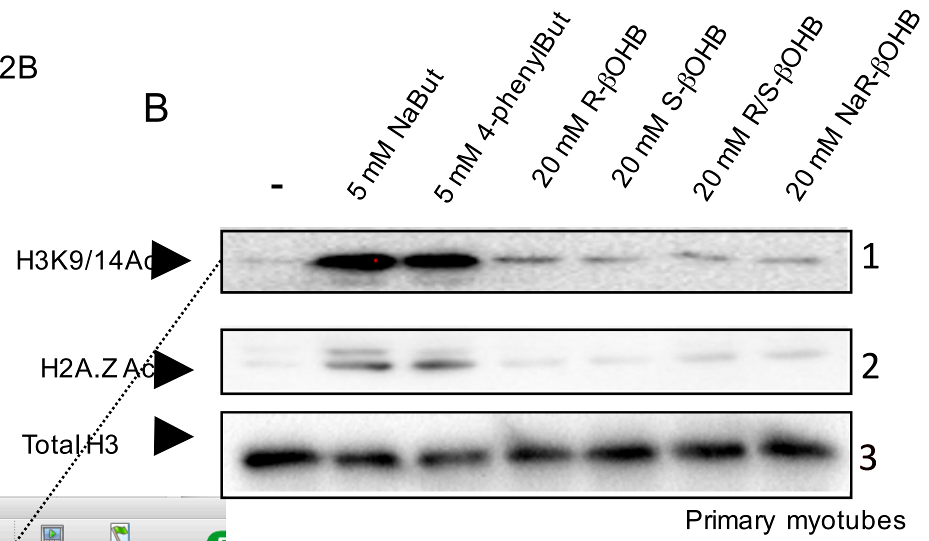

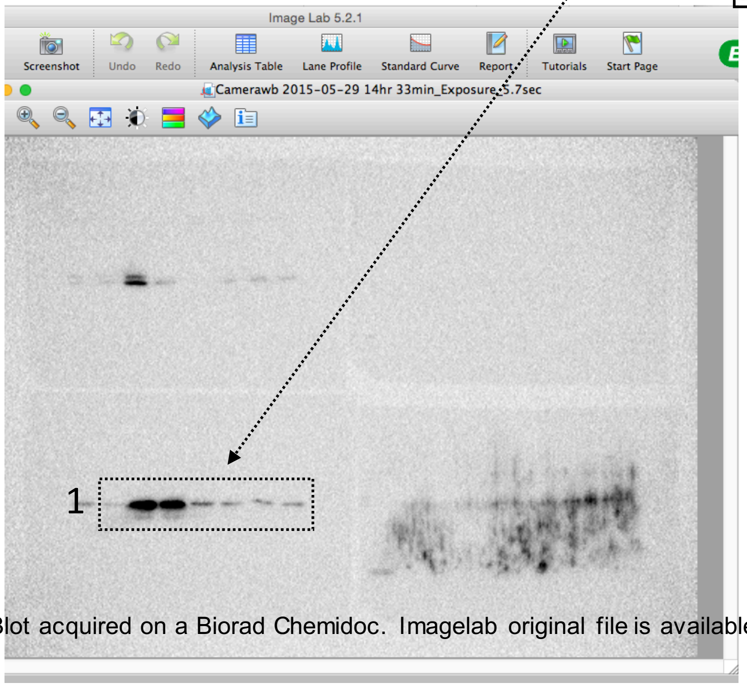

Blot acquired on a Biorad Chemidoc. Imagelab original file is available.

Chriett et al., Supplementary Figure 2
Original scans of supplementary figure 2B

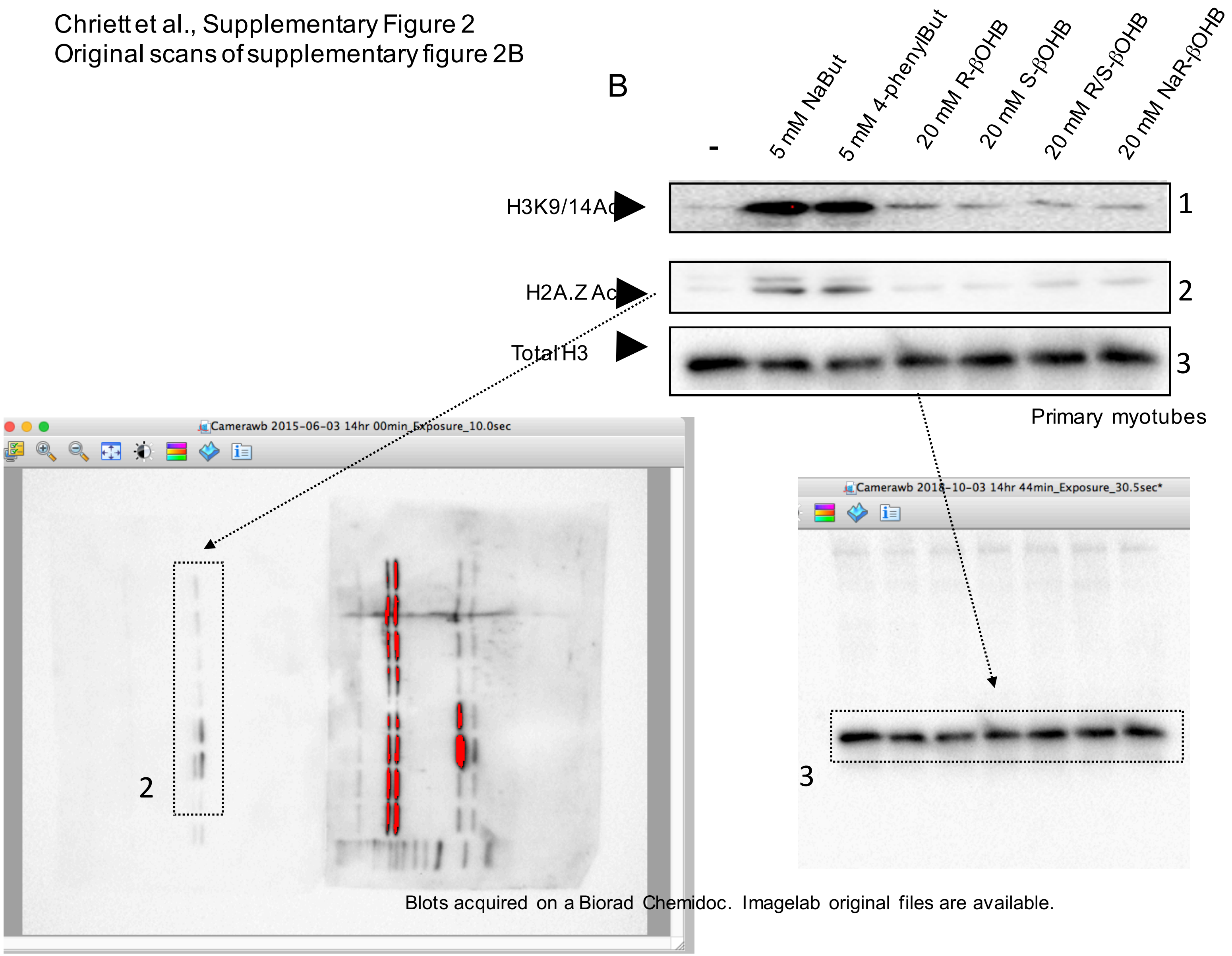

Blots acquired on a Biorad Chemidoc. Imagelab original files are available.
